# Oxy-reductive C-N bond formation via pulsed electrolysis

**DOI:** 10.1038/s41467-025-63450-x

**Published:** 2025-08-29

**Authors:** Yuxuan Zhang, Hasan Al-Mahayni, Pedro M. Aguiar, Daniel Chartrand, Morgan McKee, Mehdi Shamekhi, Ali Seifitokaldani, Nikolay Kornienko

**Affiliations:** 1 Department of Chemistry, Université de Montréal, Montréal, QC Canada; 2 Department of Chemical Engineering, McGill University, Montréal, QC Canada; 3 Institute of Inorganic Chemistry, University of Bonn, Bonn, Germany; 4 Department of Physics, Concordia University, Montréal, QC Canada

**Keywords:** Electrocatalysis, Catalysis

## Abstract

Co-electrolysis of CO_2_ with simple N-species is an appealing route to sustainable fabrication of C-N bond containing products. A prominent challenge in this direction is to promote the C-N coupling step in place of the established CO_2_ reduction pathways. This can be particularly difficult when relying on solution-based species (e.g., NH_3_) to intercept intermediates that are continually being reduced on heterogeneous catalyst surfaces. In light of this, we introduce oxy-reductive pulsed electrocatalysis as a tool for C-N bond formation. The reaction routes opened through this method involve both partial reduction and partial oxidation of separate reactants on the same catalyst surface in parallel to co-adsorb their activated intermediates proximal to one another. Using CO_2_ and NH_3_ as model reactants, the end result is an enhancement of selectivity and formation rates for C-N bond containing products (urea, formamide, acetamide, methylamine) by factors of 3-20 as compared to static electrolysis in otherwise identical conditions. An array of operando measurements is carried out to pinpoint the key factors behind this performance enhancement. Finally, the oxy-reductive coupling strategy is extended to additional carbon and nitrogen reactants and is further applied to C-S coupling.

## Abstract (web summary)

To enable electrochemical C–N bond formation, the authors have developed an oxy reductive synthetic strategy. Using a pulsed voltage sequence, partial reduction and partial oxidation of separate reactants onto the same catalyst surface promote their coupling.

## Introduction

Due to society’s ever-increasing energy demand and resultant environmental degradation from energy consumption, developing sustainable synthetic techniques to replace the fossil-fuel-driven industry is rapidly gaining interest^1^. Among such routes is electrocatalysis, which often entails lower CO_2_ emissions relative to established thermochemical routes to produce the same chemical species^2^. As a key method within chemistry’s toolbox, electrochemistry offers routes to precise reaction control by adjusting applied potentials and is thus gaining popularity as a means of carefully controlling reaction pathways towards a desired product. This is particularly important for reactions involving the transformation of CO_2_ into valuable products as multiple competing reactions could occur in parallel under typical reaction conditions^3^.

To date, electrosynthetic systems for water electrolysis are technologically mature and systems entailing CO_2_ reduction (CO_2_R) are rapidly growing as well^4–6^. However, the electrosynthetic fabrication of other classes of societally valuable chemicals are still underdeveloped^7,8^. As one of the most important classes of fertilizers and high-demand chemicals, products containing C-N bonds such as urea, amides, and amines play a pivotal role in society^9^. At the industrial level, the construction of C-N bonds from simple reactants is carried out under harsh thermochemical conditions and finding analogous electrosynthetic routes under mild conditions for these reactions is a challenge for the scientific community. The electrochemical formation of C-N bonds is further appealing as it can direct begin with readily available small molecules like CO_2_ and nitrite^10^, nitrate^11,12^, ammonia^13^, and even N_2_^14^ to directly produce value-added C-N bond products. In this context, the C-N bond coupling reaction is primarily carried out through the reduction of CO_2_ to form an activated electrophilic intermediate and subsequent attack by nucleophilic N-containing species^8^. Consequently, the selectivity for C-N bond products reactions rely heavily on the interplay of chemical C-N coupling steps and electrochemical CO_2_R pathways.

So far, most efforts in the design of electrosynthetic systems, including those for C-N bond formation, have focused on either applying a constant reductive^9^ or oxidative^15,16^ potential, where the electrode polarity remains unchanged and electrons flow unidirectionally. The crossover of oxidation or reduction products to the opposite electrode here is typically undesired^17^. Although counterintuitive, we propose that the simultaneous coexistence of oxidative and reductive intermediates on a single catalyst would be an effective route promote heteroatom coupling electrosynthesis via pulsed electrolysis.

**Fig. 1:**
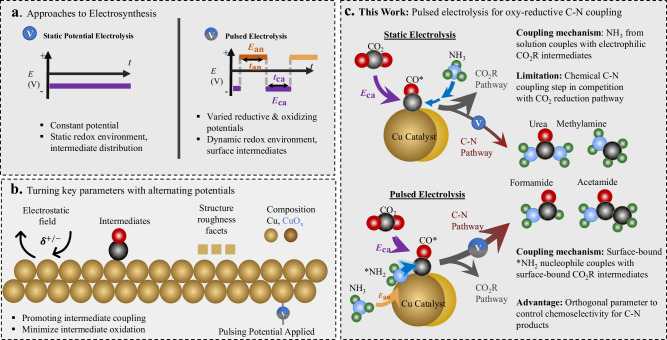
General concept of synthetic approach. Types of waveforms in static potential and pulsed electrosynthesis (**a**) and brief illustration of parameters affected through electrochemical pulsing (**b**). Our route to C-N bond production through oxy-reductive coupling via pulsed electrolysis (**c**).

Pulsed electrolysis, the application of alternating potentials has garnered interest in the CO_2_R community as this technique can afford a rational route to modulate product selectivity, stability, and activity (Fig. 1a)^18^. Within heterogeneous electrocatalysis, this technique is mainly used to modulate the catalyst surface and composition as well as the coverage of partially reduced (in the case of CO_2_R) intermediates and population of near-surface species^19–21^. In the context of organic electrosynthesis, the application of pulsed or alternating potential has led to substantial progress in several areas. For example, pulsed electrolysis enhanced adiponitrile synthesis through balance of mass transport and reaction rates at the electrode diffuse layer^22^, the rapid alternating current technique was instrumental in suppressing slower chemical reactions to attain higher selectivity in the chemoselective reduction of carbonyls^23^, and alternating currents enhanced Ni-catalyzed^24^ and Cu-Catalyzed^25^ cross coupling by periodically shifting between oxidation and reduction processes at the same electrode. However, the concept of synergistic reduction and oxidation of small molecule reactants (e.g., CO_2_, NH_3_…) onto a catalyst surface to promote their coupling has not been applied to heterogeneous electrocatalysis.

In the domain of heterogeneous catalysis, the community’s understanding of how pulsing impacts underlying physicochemical processes in the dynamic microenvironment near the catalyst surface is still being developed (Fig. 1b)^26^. To this end, operando techniques, those performed as the reaction is occurring, help elucidate the identity of catalyst phase and reaction intermediate coverage^27–29^. In this work, we make use of the synergy between pulsed electrochemistry and operando techniques (X-ray diffraction (XRD), Raman, infrared (IR) spectroscopy) to select conditions to modulate the reaction intermediate coverage during our electrosynthetic reaction to promote C-N coupling. We use Cu nanoparticles as a model catalyst, CO_2_ as the C-reactant and NH_3_ as the N-reactant to carry out C-N coupling. We select optimized conditions in which we maintain a steady-state coverage of *NH_2_ while maintaining Cu in the metallic state and show how pulsed electrolysis increased both the formation rate and selectivity for C-N products of urea, formamide and acetamide by factors of 3–20 (Fig. 1c). The synthetic value brought by this new approach is further demonstrated through the expansion of scope to additional C- and N- reactants and to C-S coupling.

## Results

**Fig. 2:**
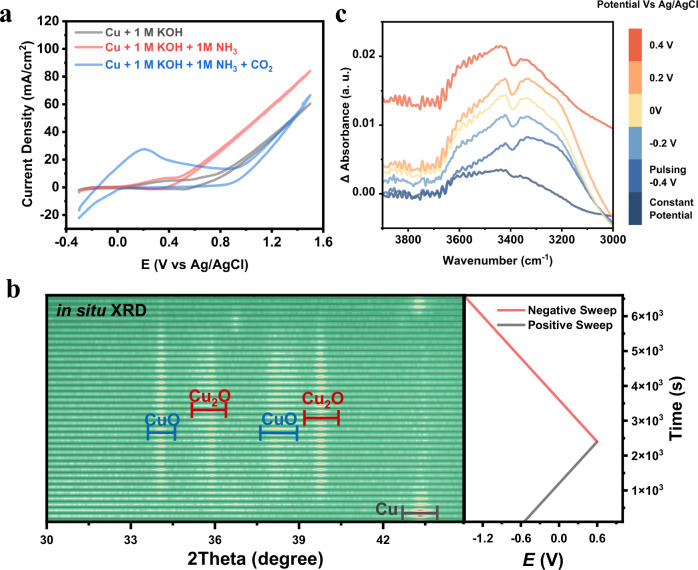
Investigation of surface coverage and catalyst transformations. CVs of the Cu showing the oxidation of Cu, NH_3_ and CO_2_R intermediates (**a**). In situ XRD similarly pointed to the dominant phase of Cu as a function of potential (**b**) while IR measurements indicated a steady-state coverage of *NH_2_ during electrochemical pulsing (**c**). All measurements were conducted in 1 M KOH and 1.5 M NH_3_ unless otherwise stated.

We carried out our measurements in a modified gas-diffusion electrode (GDE) half cell (Fig. S1), using commercial Cu nanoparticles as the catalyst and 1 M KOH as the electrolyte. Cu was chosen as a model catalyst because of the metal’s intermediate binding strength with CO_2_R intermediates^30^. This enables Cu to readily form more highly reduced, complex products from CO_2_ and these moderately-bound intermediates may also participate in C-N bond forming reactions. We first used cyclic voltammetry (CV) to identify the potentials at which various reaction steps take place (Fig. 2a). Under an N_2_ environment, the CV exhibits two distinctive anodic peaks at 0.1 V and 0.6 V (vs Ag/AgCl), stemming from the oxidation of Cu. In the presence of 1.5 M NH_3_, an increased current at 0 V indicates the first steps of surface adsorption and a large irreversible current at 0.5 V points to catalytic oxidation of NH_3_ and its derived intermediates. In the presence of CO_2_, anodic current peaking at 0.2 V likely indicates the stripping of CO_2_R intermediates from the surface.

To monitor the state of the Cu species in real time during a CV (scan rate = 0.5 mV/s), we utilized in situ XRD as a probe of the material’s crystal structure (Figs. S2, 3). After first reducing away any surface oxides, then beginning the scan in the positive direction, the XRD data shows that indeed, the transformation of Cu to Cu_2_O begins at −0.2 V and CuO subsequently emerges at 0 V, through Cu_2_O is still the dominant phase (Fig. 2b)^31^. The catalyst is fully reduced back to Cu at −1.0 V. We next sought to get a picture of possible ammonia oxidation species during pulsed conditions. In 1 M KOH and 1.5 M NH_3_ conditions, we carried in situ IR spectroscopic measurements to detect adsorbed species on the Cu as a function of potential (Fig. S4–10). We used the spectrum at open circuit conditions (around −0.2 V) as a reference, we against which spectral changes were recorded. Under steady state conditions, ammonia was oxidized to nitrite, nitrate, and hydroxylamine, beginning at 0 V as characteristic bands attributed to these species emerged in the spectra (Fig. S6). However, under pulsed electrochemical conditions (*E*_ca_ = −1.8 V, *E*_an_ varied, *t* = 1 s), peaks in the 3000–3600 cm^−1^ range emerged, characteristic of N-H bands^32^ that were not there under a constant potential of −1.8 V. We propose that these bands are indicative of *NH_2_ species that arise from partial NH_3_ oxidation that are neither oxidized all the way to nitrate nor fully reduced back to NH_3_. Further, these species may act as an activated form of nitrogen that can couple to CO_2_R intermediates via on-surface reactions as an alternative to solution-based NH_3_. This route is potentially more facile to NH_3_ diffusion from the electrolyte to react with the same CO_2_R surface intermediates.

**Fig. 3:**
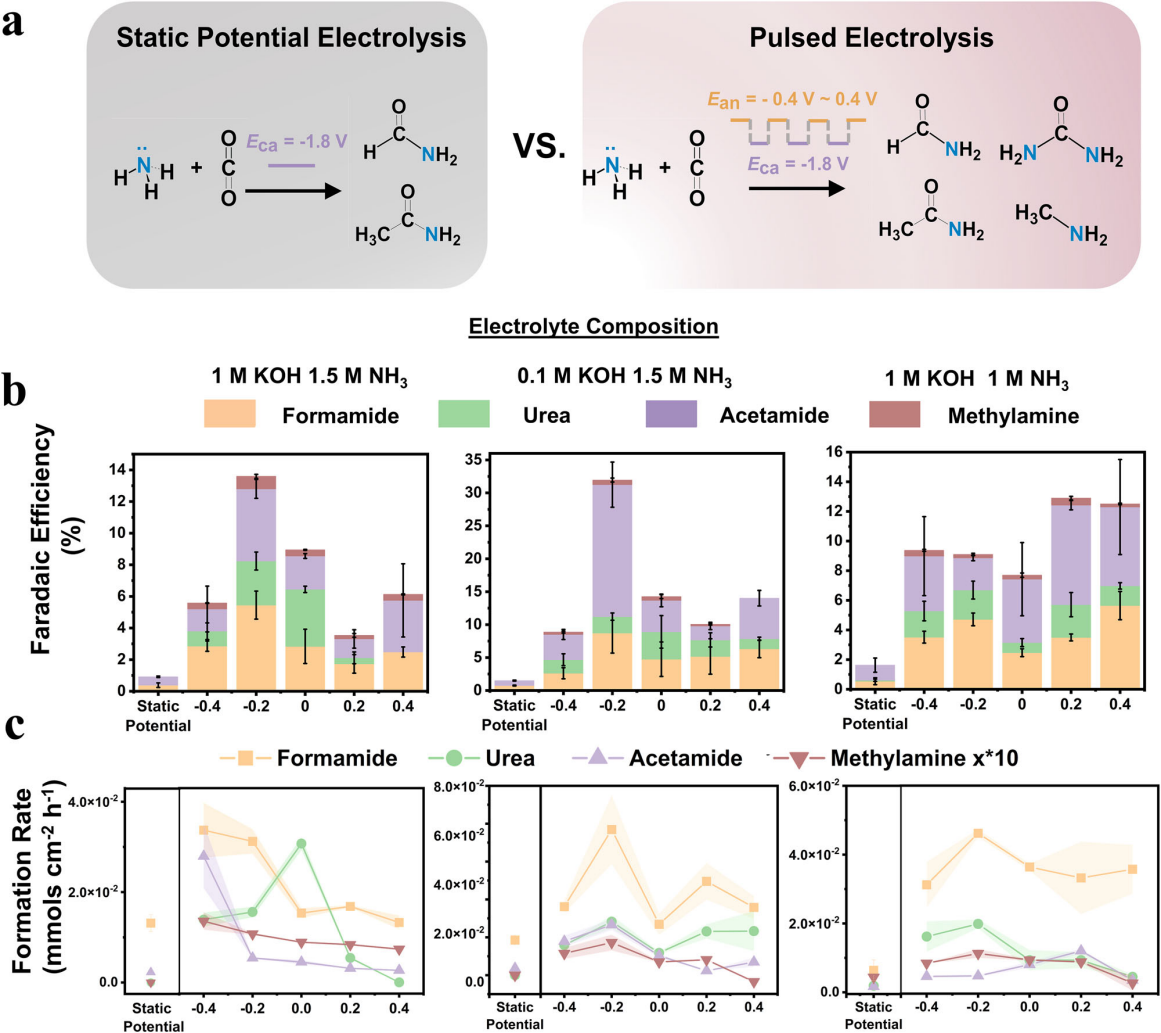
Summary of catalytic performance. Comparison of C-N products formed through steady state and pulsed electrolysis (**a**). The Faradaic efficiency (FE, **b**) and product formation rate (FR, **c**) for C-N products under three model conditions is significantly enhanced relative to that when using static potentials. For clarity, the FRs of methylamine are multiplied by factor 10. Potentials are referenced to Ag/AgCl, *E*_ca_ = −1.8 V. Error bars represent ± standard deviation from three equivalent experiments.

We next investigated the effects that electrochemical pulsing would have on the performance of the system towards electrosynthetic C-N bond formation. We used static potential electrolysis with *E*_ca_ = −1.8 V as the reference point and used systematically varied E_an_ potentials with t = 1 s for both E_ca_ and *E*_an_. Liquid C-N products were measured through NMR and their presence was confirmed through a combination of isotope labeling and 2D NMR (Fig. S14–20). While the Faradaic efficiency (FE) for C-N products was generally below 3% for the sum of C-N products under static conditions, the application of pulsing significantly increased this by factors of approx. 3–20, up to a maximum of 33% (Fig. 3b). Notably, urea and methylamine were relatively minor products under steady state electrolysis, but their selectivity significantly increased when pulsing was applied. We found that urea selectivity was maximized in 1 M KOH and 1.5 M NH_3_, while acetamide selectivity increased when 0.1 M KOH was used. Decreasing the NH_3_ concentration to 1 M maximized formamide selectivity. Methylamine was present in all conditions as a minor product. Similarly, we plotted the formation rates for all C-N products and similar enhancement rates were evident. The variation of C-N product selectivity as a function of electrolyte composition and E_an_ points to the sensitivity of the electrosynthetic system’s performance to *NH_2_ and CO_2_R intermediate coverage, Cu oxidation, local pH and double layer composition at the Cu surface.

The maximum current partial densities in this work reach only 6.4 mA/cm^2^ and 12.8 mA/cm^2^ (when only considering the cathodic pulse) for formamide and acetamide, respectively. Thus, increasing reaction throughput, alongside of selectivity, should be a central focus en route to developing economically viable electrosynthetic systems. We can point to previous C-N coupling systems that have made progress in this direction by using methanol or formate as reagents^15,33,34^.

**Fig. 4:**
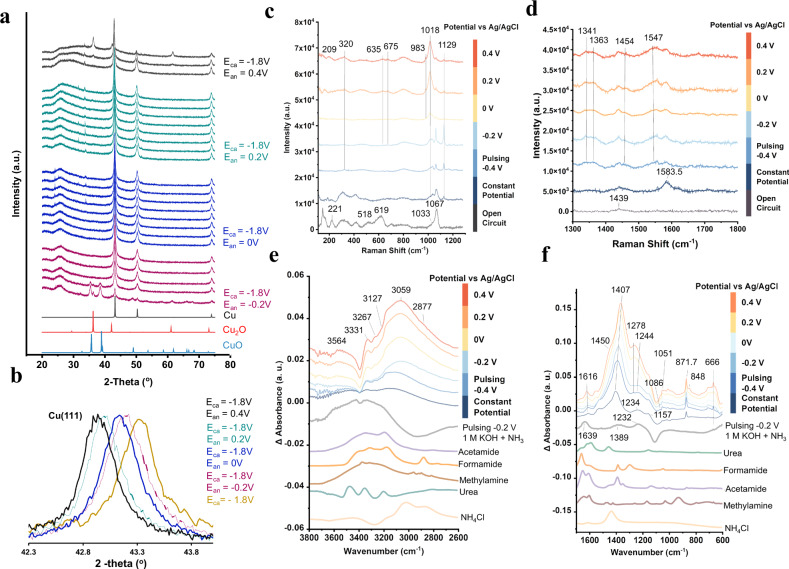
Investigation of reaction mechanisms. X-ray diffraction indicates Cu as the dominant phase during catalysis until *E*_an_ = 0.4 V, when Cu_2_O co-exists (**a**). A tensile strain was evident from the shift of the Cu (111) peak to lower 2θ values (**b**). Raman (**c**, **d**) and IR (**e**, **f**) are utilized to detect the surface bound intermediates built up under reaction conditions. All measurements were conducted in 1 M KOH and 1.5 M NH_3_ unless otherwise stated.

Next, the system was probed under typical catalytic conditions (1 M KOH, 1.5 M NH_3_) to visualize the interplay of catalyst phase and surface reaction intermediates potentially responsible for C-N bond formation. In this experiment, we began with the Cu particles as deposited and used the *t*_ca_ = *t*_an_ = 1 s as pulse durations. XRD spectra, taken at 5-min time intervals after setting a pulsing potential, show that surface oxides are reduced away within 10 min at −1.8 V (Fig. 4a). The only detectable phase is metallic Cu until *E*_an_ = 0.4 V at which Cu_2_O begins to grow. This in contrast with the results in Fig. 2b which illustrate the Cu_2_O begins to form at −0.2 V under non-pulsed conditions. This experiment indicates that Cu is the predominant active material bulk phase for this reaction and that the pulsing procedure suppresses bulk oxide formation up to 0.4 V. However, as a pulsed potential was applied, a tensile strain of up to 1.0% was evident as the Cu(111) peak increasingly shifted from 44.3 to 43.9*°* as *E*_an_ was set more positive, which may have a secondary effect on the Cu catalytic properties^35^.

Raman spectroscopy was next utilized to visualize reaction intermediates and near-surface species^27^. Similarly, Cu_2_O was reduced away when negative potentials were applied, and bands attributed to Cu-X species were noted (320 cm^−1^) (Fig. 4c). Cu_2_O reappeared at *E*_an_ = 0.2, along with bands at 530, 635 and 675 cm^−1^ attributed to Cu-OH and Cu-O_ad_^36,37^. The appearance of Cu_2_O at *E*_an_ = 0.2 V in the Raman spectra (more surface-sensitive) prior to its appearance in the XRD spectra (more bulk-sensitive) at *E*_an_ = 0.4 V is indicative of an initial surface oxidation prior to a bulk transformation, at least in part, to Cu_2_O, and under these conditions, there is likely a co-existence of Cu, Cu-OH and Cu_2_O on the surface^38^. As *E*_an_ is progressively made more positive, the CO_3_^2−^ signals (band at 1067 cm^−1^) progressively diminishes and the HCO_3_^−^ signals (bands at 1018 and 1341 cm^−1^) grow, reflective of a pH decrease at the surface^39^. Bands at 983 and 1129 cm^−1^ may indicate *CH_x_O species as they have been previously assigned at these frequencies^40^. At the higher frequency region, *CO_2_^−^ was noted (1540, 1584 and 1383)^41,42^ while new bands arising at 1454 cm^−1^ could stem from COOH vibrational modes as they typically fall within this spectral range (Fig. 4d). The peak at 1547 cm^−1^ may originate from C-N bond vibrational modes, as this peak shifts to 1512 cm^−1^ when ^15^NH_4_Cl is used to replace the ^14^NH_4_OH (Fig. S11).

Complementary to this, IR experiments showed new bands in the N-H region in addition to those from *NH_2_ when both CO_2_ and NH_3_ were present, particularly when pulsed potentials were applied (Fig. 4e). These bands roughly match the N-H vibrations of the C-N products produced and thus are indicative of enhanced C-N bond formation through pulsing. In the absence of CO_2_, bands relating to NO_3_^-^ (1389 cm^−1^) and NO_2_^−^ (1232 cm^−1^) are seen, stemming from the oxidation of NH_3_. Similarly, once CO_2_ was introduced, pulsed conditions gave rise to new bands at 1051, 1278, 1450 and 1616 cm^−1^ arising from the possible emergence of C-N products (Fig. 4f). Bands below 900 cm^−1^ may stem from Cu-C/N/O modes and several new bands in this region upon pulsing would be consistent with the existance of partially oxidized surface intermediates in combination with partially reduced CO_2_R products.

In summary, we have the following key points from our operando measurements:

1. The Cu is primarily in its metallic from until E_an_ reaches 0.2 V, at which point a surface oxide begins to form and at 0.4 V crystalline Cu_2_O domains begin to form. However, at the optimized *E*_an_ of −0.2 V, the Cu is primarily in its metallic form.
2. *E*_an_ = −0.2 V also seems to be the optimum potential because at this regime, we effectively bind *NH_x_ to the catalyst surface but not yet substantially overoxidize this species to NO_2_^−^ and NO_3_^−^ species.
3. In addition to *NH_2_ and CO_2_-derived species like *CO_2_ and *COOH on the Cu surface, we see that under optimal conditions there is a surface pH of approx. 10-11 and adsorbed O_ad_ and *OH on the surface which may also play a role in catalysis.

We note that a multitude of different reaction pathways may be in place and involved in the formation of the C-N products we measure in the reaction solution and each of these potentially pathways may differentially contribute to the overall reaction rate. In addition, multiple surface facets of Cu are present, alongside of potential defects and these further complicate the precise identification of which catalytic site(s) differentially contribute to C-N product formation. While beyond the scope of this work, an important follow-up would entail a combined theoretical and experimental elucidation of which principal mechanisms are energetically and kinetically more favorable as well as the identification of the most active Cu (or beyond Cu) surface active sites for these reactions.

**Fig. 5:**
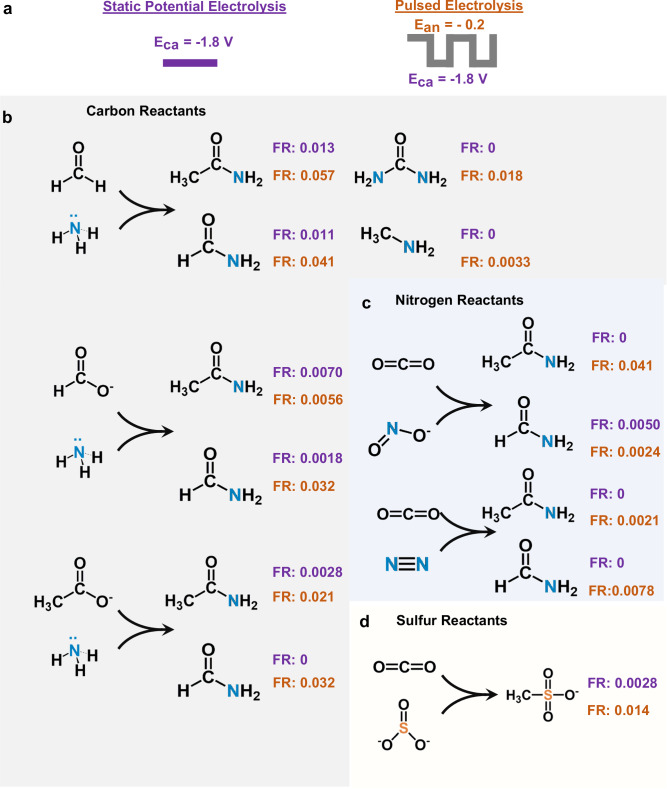
Expansion of scope. The pulsed electrochemical coupling strategy was extended to additional coupling reactions (**a**). Additional carbon (**b**) and nitrogen (**c**) reactants benefit from pulsed electrolysis to form C-N products. Finally, C-S bonds could be generated with enhanced rates in the formation of methanesulfonate (d). Formation rates are given in mMols*hr^−1^*cm^−1^ and a rate of 0 indicates that the product was not detected.

As a final endeavor, we moved to expand the scope of our measurements to show the broad applicability of pulsed electrosynthesis within the context of small molecule coupling. First, we modulated the E_ca_ and E_an_ durations under a typical set of reactions conditions. The formation rate of C-N bond products more than doubled when increasing E_ca_ to 2 s while the FE was maintained (Fig. S27). However, increasing the pulse duration of both anodic and cathodic pulses decreased the formamide production in particular, possibly due to a lower availability of *CO as the carbon pre-coupling precursor (Fig. S28). Next, we used a series of additional reactants in place of CO_2_ or NH_3_ and compared the FE and formation rate (FR), given in mMol*sec^−1^ cm^−2^ under static and pulsed conditions (Fig. 5a). Using formaldehyde (0.5 M), formate (0.2 M), or acetate (0.2 M) and coupling to NH_3_ (1 M) in 1 M KOH led to C-N bond products with significantly enhanced FR under pulsed conditions as compared with constant potential synthesis (Fig. 5b). An advantage here is that the oxidative pulse may also activated partially reduced C-species and not just NH_3_. If 0.2 M NO_2_^−^ was used in place of NH_3_, significantly increased FR were also noted for formamide and acetamide (Fig. 5c). As NO_2_^-^ can also be oxidized to NO_3_^−^, the anodic pulse may likewise help concentrate it next to partially reduced CO_2_R intermediates to promote C-N coupling. A similar route may be taking place in N_2_ activation. Finally, SO_3_^2−^, 0.2 M a simple oxidizable sulfur species, was shown to couple with CO_2_ to form methane sulfonate, a useful molecule in organic synthesis, with increased performance under coupling conditions (Fig. 5d). The coupling step here may similarly involve a nucleophilic attack by the S-species on an activated CO_2_R intermediate^43,44^. While the initial set of results here are promising, we note that the precise operating conditions were not optimized for the each experiment and there is therefore much room to grow in terms of performance. Finally, we cannot unambiguously rule out other effects that may be the dominant factor behind the enhanced reactivity such as changes to the catalyst structure induced by pulsing as the detailed examination of each reaction is beyond the scope of this work.

## Discussion

In all, this work shows how pulsed electrolysis is a powerful tool in promoting electrocatalytic coupling reactions. In particular, the partial oxidation of NH_3_ leads to a higher concentration of NH_x_ species on the catalyst surface and thereby facilitates their coupling to CO_2_R intermediates in a new reaction mechanism and consequently greater efficiencies for C-N product generation. While CO_2_ and NH_3_ coupling was the model reaction in this study, we have shown that there are many avenues to explore in the use of downstream CO_2_R products, additional N-species and even C-S coupling. Finally, there is much room to explore in terms of catalyst design. Though this work used commercial Cu particles, the use of Cu with well defined facets or Cu-based alloys may be an effective strategy for precisely modulating the binding energy of key reactants and steering the reaction down a select pathway. In addition, the addition of secondary binding sites that, for example, bind N-species may stand to boost performance. Finally, there remain many avenues to pursue to garner a more comprehensive understanding of pulsed electrosynthesis. For example, understanding how the Cu catalyst behavior is affected through voltage pulsing or utilizing complementary analytical methods like electron paramagnetic resonance to visualize radical-containing intermediates would add important pieces to the puzzle. In the end, with this newfound oxy-reductive coupling strategy, the electrochemical construction of a wide gamut of important chemicals from simple building blocks is closer to practicality.

## Methods

### Chemicals

Ammonium hydroxide solution (30–33% NH_3_ in H_2_O), Methylamine solution (40 wt% in H_2_O), Deuterium Oxide (99.8 atom% D), Ammonium-^15^N chloride (≥99.8 atom%, 15 N ≥ 99 %) Sodium sulfite (≥98%), sodium nitrite solution (40 wt% in H_2_O) was got from Sigma-Aldrich Company. Acetamide (^15^N, 98%+) was purchased from Cambridge Isotope Laboratories, Inc. Formamide, deionized (ultra pure) VMR Life Science. Copper nanopowder, APS 20–50 nm and Potassium hydroxide (flake, 85%) were obtained from Thermo Scientific. Carbon cloth (ELAT LT 1400W-40 × 40 cm) was purchased from FuelCellsEtc. Nafion D-521 dispersion (5% w/w in water and 1 propanol, ≥0.92 meq/g exchange capacity), Sodium formate 98 % were obtained from Alfa Aesar. Ethanol, 2 propanol, Methanol (HPLC grade) were got from Fisher chemical company. Acetic Acid were purchased from MACRON fine chemicals. Formaldehyde solution 37% was obtained from Ward’s science. Sodium methanesulfonate was obtained from TCI America.

### Electrode preparation and characterization

The microstructure and composition of electrode after electrolysis were investigated by transmission electron microscopy (TEM) and XRD.

Transmission Electron Microscopy and elemental mapping: TEM images were collected at The Facility for Electron Microscopy Research of McGill University. TEM characterization was performed using Thermo Scientific Talos F200X G2 (S)TEM with High visibility low-background beryllium double-tilt optimized for energy-dispersive X-ray spectroscopy (EDS). All samples were prepared by carefully scratching off from the electrode and dispersing them onto ethanol solution. After sonicating for 5 min, the solution was drop cast onto a copper grid supporting a thin electron transparent carbon film. High angular annular dark-field imaging (HAADF) performed in parallel with EDS acquisition in the collection angle of 58–200 mrad.

XRD patterns were collected in the range of 10° ≤ 2θ ≤ 80° on a Panalytical MPD-PRO diffractometer equipped with a linear X’celerator detector with CuKα (1.5406 Å) anode. See Supplementary Note 3 for details.

### Electrochemistry analysis and product quantification

Electrochemistry experiments were carried out in a home-made GDE cell. A modified GDE half cell was used to maximize the sensitivity of the measurements through the use of lower electrolyte volumes. A carbon cloth loaded copper catalysts is sealed in the middle of a sandwiched structure. CO_2_ molecules can transfer from the bulk gas phase to the gas-liquid boundary layer through the bottom layer (constant 10 mL/min). NH_3_ was fed through the liquid phase (NH_4_OH) with different concentrations dissolved in 1 M KOH solution. For a typical electrolysis experiment, 1 mL electrolyte was added into the hydrophobic layer of carbon cloth electrode and saturated with CO_2_ for at least 5 min. The reference (Ag/AgCl gel in saturated KCl, 25 °C) was used to calculate RHE with *E*_RHE_ = *E*_Ag/AgCl_ + 0.059 pH + *E*°_Ag/AgCl_, where *E*°_Ag/AgCl_ = 0.206 V. The Ag/AgCl reference was periodically checked against a master reference electrode for any potential drifts to maintain stable in alkaline electrolyte. Prior to CO_2_ bubbling, the electrolyte was nominally pH 13.7 (1 M KOH) or 13.0 (0.1 M KOH). As CO_2_ was bubbled and the electrode commenced, the pH, especially near the surface decreased to a value of 10-11.

Electrochemical measurements were conducted through a Biologic SP200 potentiostat and EC-lab software v11.43. A graphite rod was used as the counter electrode. Electrolytes were prepared by mixing de-ionized water and the requisite salts and stored in under ambient conditions until use.

Prior to electrochemical measurements, the impedance between reference and working electrode was recorded at open circuit (100 KHz) and the ohmic drop was subsequently corrected for at 85% with the ZIR function in the EC-lab software. The resistance values were typically on the order of 1.5 ± 0.4 ohms. The area used to normalize current density in the CV plots is the geometric surface area, using a consistent mass loading of 10 mg of Cu. The electrode area was 0.8 cm^2^.

The static electrolysis for typical chronoamperometric measurements is conducted at −1.8 vs Ag/AgCl (denoted as *E*_ca_). The reported results were obtained after 1800s under CO_2_RR. For the experiments involving pulsing electrolysis, three techniques were applied: (1) one chronoamperometric run at the anodic potential (*E*_an_) for 1 s, (2) cathodic chronoamperometric (*E*_ca_) for 1 s, and (3) 899 loops of (1) and (2). The pulsing anodic potential in our study was varied between −0.4 V to 0.4 V, with a 0.2 V interval. This range of potentials was selected based our analysis, which presents in the paper. Next, the cathodic reduction time and anodic time was varied to 2 s, to test the influence of the anodic-cathodic time ratio.

The gas products reported were measured by gas chromatography (SRI Model 8610C GC) equipped with a thermal conductivity detector and flame ionization detector. The gas flow rate was kept constant at 10 mL/min as measured by a flow meter (Dwyer Instruments, Inc) at the exit of the GDE cell. Ultra-high purity nitrogen gas was used as the carrier gas. The electrochemical cell was directly connected to GC, therefore during reaction the CO_2_ continuously flowed through the reactor and GC. The gas was sampled from the reaction vessel 30 min after applying the reducing potential. Calibration gases at various dilutions were used to establish a calibration curve for accurate product detection.

Liquid products were measured by NMR (AV NEO 400, Bruker BioSpin). In the present study, long-term electrolysis runs over 6 h are conducted in pulsed mode. Time can not be prolonged for more because of the flooding of the carbon cloth electrode. To prove C-N bond formation in the pulsed electrolysis, the electrolyte after 6 h electrolysis was analyzed on a GC 7890 A coupled to an MS G3174A (Agilent technologies). Faradaic efficiencies were calculated by dividing the electrons that go through the circuit towards a particular product by the total charge that passed through the circuit. FE measurements are given as the average ± standard deviation from 3 independent measurement. Formation rate in Fig. 5 are single measurements.

## Supplementary information

Supplementary Information

Transparent Peer Review file

## Source data

Source Data

## Data Availability

All data will be available upon request to the corresponding author via email for non-commercial purposes. Data will be saved for 10 years, and requests will be responded to within 10 working days. Data corresponding to the main text and supplementary information are deposited alongside the manuscript as source data files. Source data are provided with this paper.
