# Supplementary material for: Oxy-reductive C-N bond formation via pulsed electrolysis
Source: Nat Commun. 2025 Aug 29;16:8106. doi: 10.1038/s41467-025-63450-x (PMC12397372; doi:10.1038/s41467-025-63450-x)
Supplement: Supplementary file 1 — Supplementary Information [file 41467_2025_63450_MOESM1_ESM.pdf]

Supporting information for:

Oxy-reductive C-N bond formation via pulsed electrolysis

Yuxuan Zhang[1], Hasan Al-Mahayni[2], Pedro M. Aguiar[1], Daniel Chartrand[1], Morgan McKee[1,3], Mehdi Shamekhi[4], Ali Seifitokaldani[2]* and Nikolay Kornienko[1,3]*

[1]Department of Chemistry, Université de Montréal, 1375 Ave. Thérèse-Lavoie-Roux, Montréal, QC H2V 0B3

[2]Department of Chemical Engineering, McGill University, 3610 University Street, Montréal, H3A 0C5 Québec, Canada

[3]Institute of Inorganic Chemistry, University of Bonn, Gerhard-Domagk-Str. 1, 53121 Bonn, Germany

[4]Department of Physics, Concordia University, 7141 Sherbrooke St. W., Montréal, QC H4B 1R6, Québec, Canada

*Correspondence to: ali.seifitokaldani@mcgill.ca and nkornien@uni-bonn.de

Supplementary Notes

Supplementary Note 1: Using NMR to detect C-N products.

The development of electrochemical C-N bond formation via activating small molecules is hampered by the lack of effective methods for quantitatively detecting the C-N bond products. Colorimetric quantification methods such as the reaction of urea with diacetyl monoxime[1,2] and enzymatic methods[3,4] are heavily relied upon for C-N bond detection, especially for urea. Those methods are well-established in aqueous media, but it is notoriously sensitive to variations in electrochemistry, multiple reaction agents, and tedious procedures in sample preparation are inevitable. The colorimetric method is significantly hindered in these media by the interference caused by temperature, pH, and ion concentration[1,4-6]. A number of advanced analytical techniques are capable of measuring urea such as Mass Spectroscopy; however, the separation of product and electrolytes is necessary due to the instrumental requirements[7,8]. Furthermore, the analysis and quantification the product requires NMR or HPLC to analyze the remainder of the CO_2-related liquid products.

Therefore, robust, and general methods of C-N analysis are needed. To this end we utilised proton nuclear magnetic resonance spectroscopy (^1H NMR). In contrast to indirect spectrophotometric detection after derivatization, 1H NMR provides direct and highly selective analysis of ^{14}N-related species and ^{15}N-related species[9]. Moreover, NMR allows for simultaneous detection and quantification of conventional CO_2 reduction products as well as the C-N bond related products, resulting in a more efficient product analysis.

However, the detection of hydrogens directly bound to nitrogen by NMR requires care in sample preparation owing to their exchangeable nature (function of pka) and due to the intrinsic nuclear properties of the dominant isotope of nitrogen at natural abundance, ^{14}N (N.A 99.63%). ^{14}N has spin, I > ½ (I=1) and thus, in addition to a nuclear magnetic moment (observed for all nuclei with non-zero nuclear spin, I) it has a non-zero nuclear electric quadrupolar moment (Q). This latter interaction can induce severe line-broadening to the nitrogen NMR signal itself, as well as to the signals of nuclei attached to the nitrogen. The impact of the linewidths decreases as local symmetry about the nitrogen increases and is lowest for cubic symmetry ($T_{d,}$; e.g, NR_4 species). Species with higher symmetry will yield sharper ^{14}N and 1H NMR peaks. Thus, working at pH where e.g, RNH_2 is present predominantly as RNH_3^+ or NH_3 is present as NH_4^+ permits easier acquisition of 1H NMR of such species. At pHs above the pka the rate of exchange of NH hydrogens with other exchangeable hydrogens such as those of water can make these signals invisible due to exchange induced broadening.

Therefore, without turning the pH of electrolyte after reaction, sub-optimal NMR settings can result in lengthy data acquisition times[10] and underestimated quantification. In addition, formamide and urea are only stable at certain pH range. The formamide would discompose into formate either in low pH or high pH, which is typical of conditions employed in many experiments in the literature targeting C-N bond formation[11,12]. The drop of pH in alkaline conditions and the formation of buffer pairs of CO_3^{2-} /HCO_3^- during the reaction further hinders quantitative detection.

Herein, we reported a versatile method to obtain quantitative analysis of urea in high range pH electrolyte by ^1H NMR. The concentration of C-N bond products can be accurately determined in the range of a minimum of 1 mM and while minimizing C-N product decomposition. The method is promising for electrolysis applications, especially pulsing electrolysis, with the advantages of simplicity, high accuracy, and fast non-destructive detection.

DMSO (≥99% purity) was employed as an internal standard for quantitative ^1H NMR. Its distinct and intense methyl singlet does not overlap with common product peaks of CO_2 reduction, and it is miscible with a variety of solvents.

Phosphate buffer (PBS, 2.0 M) was added to each sample to adjust solution to a pH equal to 5-7 to protect the pH-sensitive C-N bond and protonated urea. If the pH of the final solution is well below the molecule's pKa, then ^1H NMR yields relatively sharp signals arsing from N-H hydrogen(s)[13]. However, if the electrolyte pH is higher than the molecule's pKa, protons would exchange with the water, generally prevents detection of N-H bonds.

A common practice in biochemistry is either to use a non-exchanging solvent such as DMSO-d6 or 1:9 D_2O:H_2O combined with water suppression techniques[14]. In our electrochemical reaction, DMSO-d6 is not chosen as the locker because its solubility of ions and products in the resultant solution is too low, although have a better N-H signal. D_2O and distilled water were added so that the final D_2O concentration was 10% (v/v; presence of small amount of deuterium in the solution has minimal impact to the urea quantitation as deuterium is distributed in urea and water in the same isotopic ratio).

All NMR samples were prepared to have a final H_2O: D_2O ratio of 9:1 by mixing appropriate volumes of electrolyte, PBS, and D_2O in 7:2:1 ratio. The method presented here detects both formamide, acetamide, and urea N-H bonds, but does not detect ammonium N-H bonds. This is because the pH of showing hydrogen attached to different nitrogen is different. The beneficial characteristic rules out the interference of the NH_3 and false positives from this species. All ^1H NMR experiments are the sum of 32 co-added scans with a 60s relaxation delay between scans to ensure all spins had relaxed to equilibrium (NB: the long relaxation time necessary was due primarily to the slow relaxation of the aldehydic hydrogen of formate/formamide).

Supplementary Note 2: Using $^{15}NH_4Cl$ to prove the formation of C-N bond.

Both Isotope labelling and ^{1}H-^{15}N heteronuclear single-quantum correlation (HSQC) experiment were conducted to prove the C-N bond product formation. The results for the aqueous solutions are in good agreement with those reported previously[9].

Table 1. Proton chemical shifts of Acetamide-^{15}N in D_2O/Buffer solution/ 0.1 M KOH = 1:2:7. All the chemical shift has been calibrate based on DMSO in our solution recipe as 2.71 to get rid of the effect of pH.

Acetamide	Acetamide - ^{14}N	Acetamide -^{15}N
δ_{CH_3}(ppm)	1.98	1.98
δ_{H_3} (ppm)	7.54	7.54
δ_{H_b} (ppm)	6.78	6.78
$\Delta\delta_{ab}$(ppm)	0.76	0.76

Table 2. A comparison of coupling constant (Hz) in the amide groups of Formamide-^{15}N and Acetamide-^{15}N.

	Formamide - ^{15}N in water	Acetamide - ^{15}N in water
$T(H_a - H_b)$ (Hz)	2.3	2.2
$T(15N - H_a)$ (Hz)	91.3	90.9
$T(15N - H_b)$ (Hz)	86.93	88.4
$\Delta\delta_{ab}$ (Hz)	0.37	0.756

Supporting Note 3. Identification of the oxidation potentials for Cu nanoparticles in the GDE cell.

Electrolytes were saturated with CO_2 for at least 15 min prior to applying voltage and conducting *in situ* experiments. See the **Figure S2. S3** for detailed set up of the operando XRD set up and cell.

Both *ex situ* XRD patterns and *in situ* XRD were collected with a Malvern PanAlytical Empyrean 3 diffractometer with a Cu Kα radiation source ($\lambda = 1.5418$ Å) and a PIXcel^{3D}detector in 1D mode operated in Bragg Brentano ($\theta - \theta$) geometry. For a typical ex situ measurement, a range of 5° ≤ 2θ ≤ 80° measured with a PIXcel3Ddetector in 1D mode configuration with iCore and dCore optics with automatic slits set at 10 mm irradiated length and a collection time of 1h.

To monitor the copper oxidation state, we tracked the crystallinity of the catalysts by means of time-dependent XRD conducted simultaneously with CV measurements. We first apply a constant potential at -1.8 V for 30 minutes under CO_2-saturated 1 M KOH with 1.5 M NH_3 electrolyte to completely remove any surface oxide species. During the CV, the potential applied to the sample was changed from -0. 6 V and + 0.6 V, and then from +0.6 V to sweeping back to -1.3 V vs. Ag/AgCl, with a rate of 0.5 mV/s. The XRD detection system used a GaliPIX3D detector in 1D mode configuration with BBHD optics with a range of 30° ≤ 2θ ≤ 45°, leading to each spectrum for 160s in total.

To mimic the real operation condition when each pulsing electrolysis operates, we track the copper nanoparticle in real time when applied to the pulsing condition. Each pulsing condition would track around 30 min as the electrolysis time we keep normally is 30 mins. The pattern was collected between 20° ≤ 2θ ≤ 75° for each spectrum of 5 min.

Supplementary Note 4*: In situ* IR analysis

Infrared spectroscopy in an ATR configuration was performed with a ThermoFischer Nicolet 380 FTIR-ATR system with a ZnSe ATR crystal coated with a diamond surface. Infrared spectra were collected at room temperature over the range of 400-4000 cm^{-1}. For ex-situ measurements, each spectrum was recorded with an accumulation of 800 scans with a resolution of 4 cm^{-1}.

For *in situ* IR spectroscopy, the experiments were executed through the use of a custom-designed spectroelectrochemical cell. The carbon cloth was placed in the middle to separate the electrolyte and the gas chambers. Simultaneously, glassy carbon rod was used as the counter electrode and the Ag/AgCl was used as the reference. Each infrared absorption spectrum was acquired by averaging 800 scans and then subtracting from the spectra at open circuit potential. The background spectrum of the catalyst electrode was acquired at open-circuit voltage before each systemic set of measurements, and the measured potential ranges of the electrocoupling reaction were set to 0.4 to –0.4 $V_{Ag/AgCl}$ with an interval of 0.2 V.

In the first part of in situ IR experiments, we aim to identify the onset potential for NH_3 oxidation by combining electrochemical analysis and in situ IR spectroscopy. In open circuit conditions, we add 1.5 M NH_4OH into the solution to see the adsorption behavior of ammonia on the copper surface.

We applied a series of constant potentials to observe the initiation of ammonia oxidation on copper, namely the dehydrogenation of the NH_4OH to absorbed $*NH_2/NH*$. From Figure S18, we can observe NH_2* peak and NH* species at the very beginning of the reaction and a transition point start from 0V, as the NO band begins to rise and indicate the oxidation of $*NH_2/NH$.

The second part of our *in situ* IR experiments involves determining if the pulsed condition would have a constant $*NH_2/*NH$ species coverage. Based on the CV results (Figure 2a), we selected a potential range of -0.4 V to 0.4 V for the anodic pulse potentials with a gap of 0.2 V. There are two possible reactions in the pulsed electrolysis in the 1M KOH, 1.5 NH_3 solution: as in the steady state anodic potential conditions, ammonia is being oxidized[16]. However, upon the application of a cathodic potential of -1.8 V, the formed partially oxidized species would be partially reduced back to NH_3[17,18]. Hence, it's important to investigate if there are still any $*NH_2/*NH$ species remaining on the copper surface.

We can observe from the Figure 2c, in the constant potential electrolysis, only NH_4^+ related peak present. In contrast with the constant potential, when applying pulsed electrolysis, peaks in the range of 3000-3600 cm^{-1} emerged, we tentatively ascribe them as the NH_2*. That being said, the steady state of partial NH_3 oxidation related species remain on the catalysts surface with select a range of pulsed electrolysis conditions.

In the third part, we aimed to conduct a series of experiments to detect C-N bond products being formed.

In the final part of the IR experiments, we maintain the E_{ca}=-1.8V and E_{an} = -0.2 V with a different time duration was conducted to elucidate the change of the spectra peak with different pulsing times.

Isotope labelling using $^{15}NH_4Cl$ to replace the $^{14}NH_4OH$ was conducted to identify bands associated with C-N bond and support our analysis.

Supplementary Note 5: Raman measurements:

In situ and *ex situ* Raman spectra were recorded with a Renishaw Raman spectrometer using a 5 mW 633 nm excitation laser and 1800 mm^{-1} grating. Before each set of measurements, detector calibration was conducted by measuring an internal Si wafer (521 cm^{-1}). A laser line focus module was utilized to obtain spectra by spreading out the laser intensity with approximately 20x lower laser intensity per area.

Spectra were collected in the range of 100-1800 cm^{-1} with an exposure time of 5 min for each acquisition. The laser power was 5 mW. An immersion objective (numerical aperture of 0.8) was used with a custom gas diffusion electrode for maximum signal intensity. These spectra analyzed and processed using WiRE 4.4 and OriginPro 2022 software. The spectra were baseline-subtracted using the polynomial feature of eight order, and cosmic rays were removed.

The *in situ* GDE cell had a liquid electrolyte reservoir in which the immersion objective was dipped, and the carbon cloth coated with copper catalysts separated the electrolyte reservoir and the gas channel. CO_2 was continuously delivered to the catalyst at a flow rate of 10 sccm. The counter electrode, a glassy carbon rod, and the reference electrode, Ag/AgCl, were dipped in the electrolyte reservoir around 0.5 cm away from the cathode. After purging the CO_2 into the electrolyte for more than 15 min, we acquired a spectrum of open circuit to observe the surface species prior to electrochemical reaction. Then, a constant potential was applied, to observe the species during the static electrolysis. In the following step, we applied pulsed electrolysis conditions one by one. Electrochemical parameters were the same as described above. 1M KOH was used as electrolyte, with the presence or absence of 1.5 M NH_4OH to identify the peaks associated with C-N bond. In addition, the different electrolysis condition (static electrolysis and pulsed electrolysis) was compared to observe the difference in surface intermediates.

In the frequency range of 1200-1800 cm^{-1}, the Raman spectra of carbon cloth overlap with some important vibration modes of the C-N bond feature, so we modified the gas diffusion layer while preserving its main function. To reduce the background spectra, carbon paper was used to support copper nanoparticles. The carbon cloth is placed on the bottom of the carbon paper catalyst support so that it maintains its hydrophobicity. As the carbon cloth is made of fibers, the interspace of the fibers can let the gas permeate, so the gas diffusion layer still serves its primary function.

In the final step, we maintain the E_{ca} = -1.8V and E_{an} = -0.2 V with a different time duration to elucidate the probe of the spectra (and surface speciation) with different pulsing times.

Isotope labelling of $^{15}NH_4Cl$ to replace the $^{14}NH_4OH$ and KOD to replace the KOH, H_2O to replace the D_2O was conducted to identify the peak associated with C-N bond, and peak associate C-H vibration mode.

To better express the features of the Raman spectra in the range of 1200-1800 cm^{-1}, we utilized OriginPro 2022 software to process the Raman spectra. The Savitsky-Golay method was conducted as a fit method and the raw data and after-processed data are plotted on the same graph.

Inspired by work using *in situ* Raman to observe of the pH gradient near the GDE electrode of CO_2 Reduction in alkaline electrolyte[19], we analysis the local surface pH on the surface of the copper nanoparticle. The local pH could determine by the ratio of CO_3^{2-} $/HCO_3^-$. During our *in situ* Raman experiment, the distance between the immersion objective and the catalysts remains constant. Therefore, we can use the peak intensity of CO_3^{2-} and HCO_3^- to roughly determine the surface pH in different electrolysis conditions. We can observe the peak intensity of CO_3^{2-} decreasing, while the peak intensity of HCO_3^- is increasing, which indicates that the surface pH will increase with different anodic pulsed potentials. Also, since in the oxidation process, the surface *OH could react with the NH_3 to form some NH_2 or NH[16], this means the consumption of OH^- during pulsed condition might occur faster than the without NH_3 condition. This may help explain why under 1M KOH and 1M NH_4OH electrolyte composition, the reaction has higher ethanol selectivity than the 1M KOH, 1.5 M NH_4OH. The increase of bicarbonate concentration may also increase the selectivity for CH_4[20].

Supplementary Note 6: Scope of oxy-reductive C-N bond coupling.

With an initial set optimized reaction parameters (for CO_2 + NH_3) in hand, we sought to explore the scope and limitations of the proposed reaction mechanism and the unique method.

Reaction conditions: Formaldehyde (CH_2O) substrate (0.5 M), NH_3 (1 M), sodium formate and sodium acetate (0.2 M), Na_2NO_3 (1 M) in the 1M KOH electrolyte. For the C-S bond coupling step, SO_3^{2-} (0.2 M) was used as the sulfur source. The CO_2 bubbling rate remained 10 sccm. When using formaldehyde, formate and acetic acid as the carbon source, N_2 (10 sccm) flowed through the electrolyte and cell for 15 min to avoid the influence of atmosphere CO_2.

The reaction mixture was electrolyzed for 30 min both in condition of static electrolysis and a pulsed electrolysis. The pulsed electrolysis condition maintained E_{an} = -0.2 V for 1s and E_{ca}= -1.8 V for 1s. However, the optimized conditions might be different for each substrate/reaction. It is beyond the scope of this work to optimize the conditions for each of the substrates, although we believe we may attain better results through screening on different potentials, pH, reactant concentration and more. Because of the uncertain reaction routes and mechanisms, the formation rate was used as a more reasonable metric to compare the static electrolysis and pulsed electrolysis.

Supplementary Note 7. Using GC-MS to prove the formation of the C-N bond products.

To further verify our results for the C-N bond formation, we conduct GC-MS analysis of the electrolyte after 6h electrolysis.

1.15 ml of 2 M Phosphate buffer was added to the electrolyte after electrolysis (around 4 mL) for pH adjustment and to avoid the possible C-N discompose. The obtained solution mixture was then concentrated using a rotary evaporator to get around 1 mL solution in a low-pressure mode. Prior to GC-MS analysis, the formamide, acetamide, and urea were extracted from a liquid/liquid extraction. An aliquot of 300 mL of the sample was mixed with 600 mL ethyl acetate and 100 mL methanol, vortexed then centrifuged.

An aliquot of 1 mL of the supernatant was injected into the GC-MS system with a split ratio of 20. The GC column was a DB-624, 30m x 0.25 mm, 1.4mm, operated under a temperature gradient starting at 40°C, held for 1 min, increased to 195°C at 10 °C /min, and then to 250°C at 10 °C /min for a total run time of 22 min. The ionization mode was EI and mass spectra were acquired in scan mode from m/z 10 to 500. A database search on the NIST05a library was used for compound identification. Besides solvent-related peaks, formamide, acetamide, and urea were identified in the sample with matching scores of 98.13, 98.64, and 96.11 respectively (see Figure SI-28, as generated by the Agilent database search software).

Supplementary Note 8. Determination of Cu catalysts dissolution using ICP-OES.

The dissolution of Cu was monitored via ICP-OES by evaluating the electrolyte after electrochemical synthesis. The initial loading of Cu on carbon cloth was 10 mg and we monitored the dissolution of copper around 2h of pulsed electrolysis condition maintained E_{an} = -0.2 V for 1s and E_{ca}= -1.8 V for 1s. The initial sample loss might contribute to part of catalysts that has loose contact with the carbon clothe. After 30 min, we can observe additional dissolution of Cu, and this is a challenge to be addressed when considering scaling up and long-term operation.

Given the detected ICP-OES data, we apply 50 mM Cu^{2+} as electrolyte to identify the effect of in situ dissolved Cu(II) to the product formation in our reaction system. In this experiment, only carbon cloth used as working electrode to apply the reaction and we used the same condition (E_{an} = -0.2 V for 1s and E_{ca}= -1.8 V for 1s). We observe that Cu is rapidly deposited into its metallic form under reaction conditions so elucidating the role of Cu ions in solution is not unambiguous. However, this experiment did not yield any C-N products within our detection range. We keep this as an intriguing open question to follow up on in future studies.

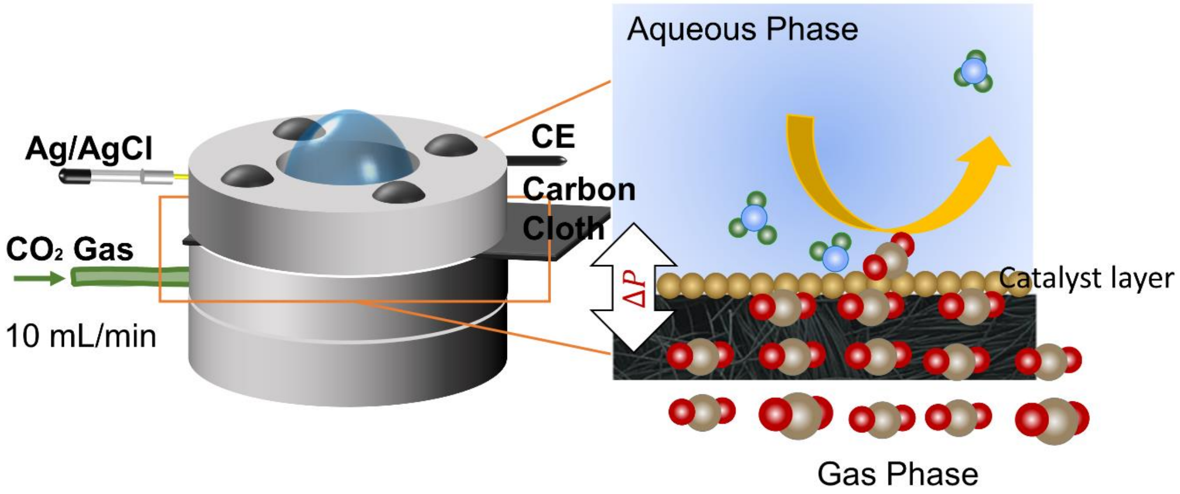

Figure S1. **Electrochemical Reactor Setup.** Simplified schematic of the electrochemical GDE based setup employed in this work. A homemade Teflon low volume GDE batch cell was used to maximize product sensitivity while enabling catalysis at a gas liquid solid interface.

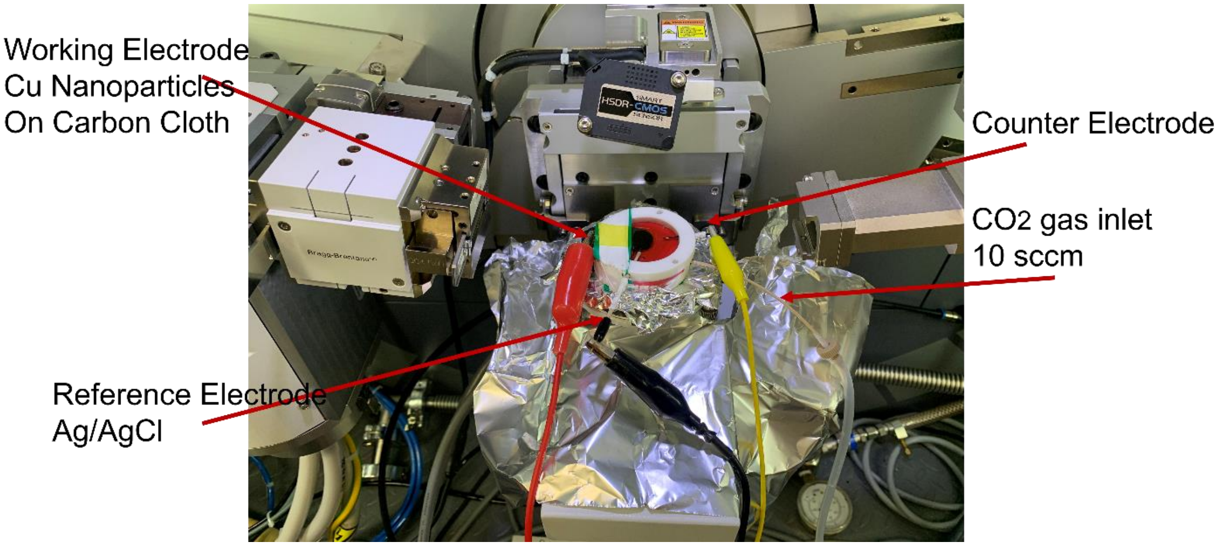

Figure S2. **XRD Measurement setup.** *Operando* cell for surface X-ray diffraction measurements in an electrochemical environment. A kapton layer was cover on the working electrode surface to avoid the vaporization of the electrolyte.

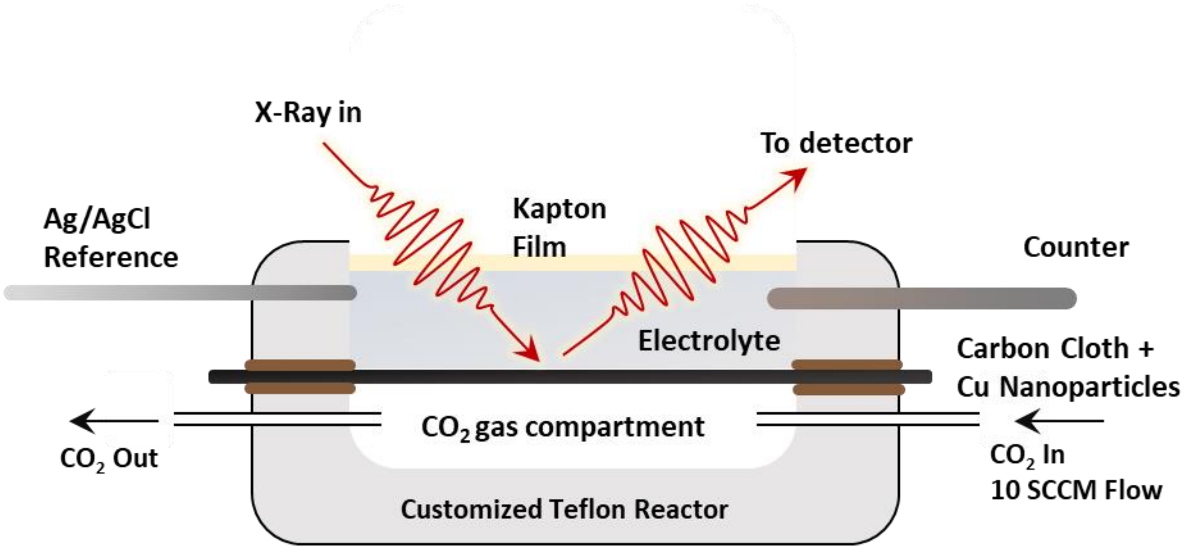

Figure S3. **Raman spectroelectochemistry setup.** Simplified schematic of the in situ XRD cell setup employed in this work. The cell was made to closely mimic the reactor used for reactivity measurement and still enabled reactivity at and probing of the gas liquid solid interface.

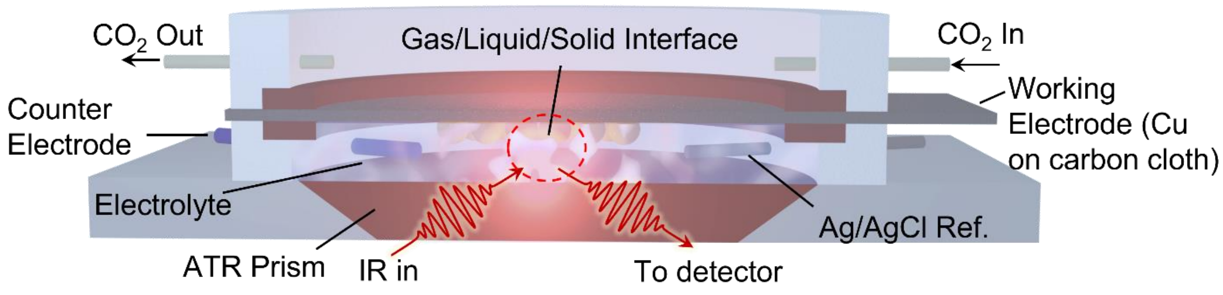

Figure S4. IR spectroelectrochemical testing. The spectroelectrochemical configuration employed to probe the reaction process is illustrated. This cell employed an upside down configuration of the gas-liquid solid interface and used a thin aqueous layer.

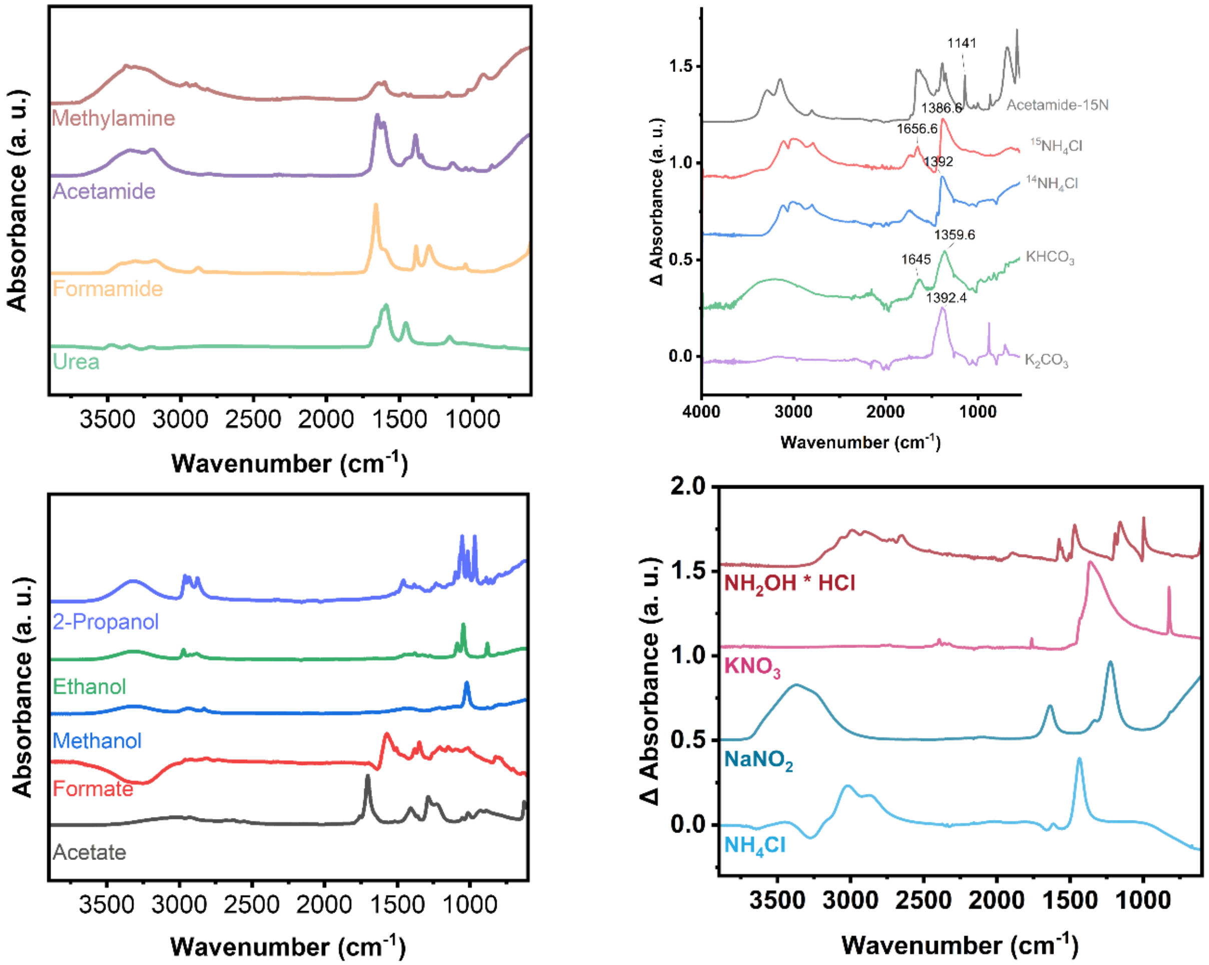

Figure S5. **IR spectra of standards**. Infrared spectra of several reference compounds. The spectra of the standards as either as pure liquids or powders were taken, otherwise they were highly concentrated in water which was used as a background.

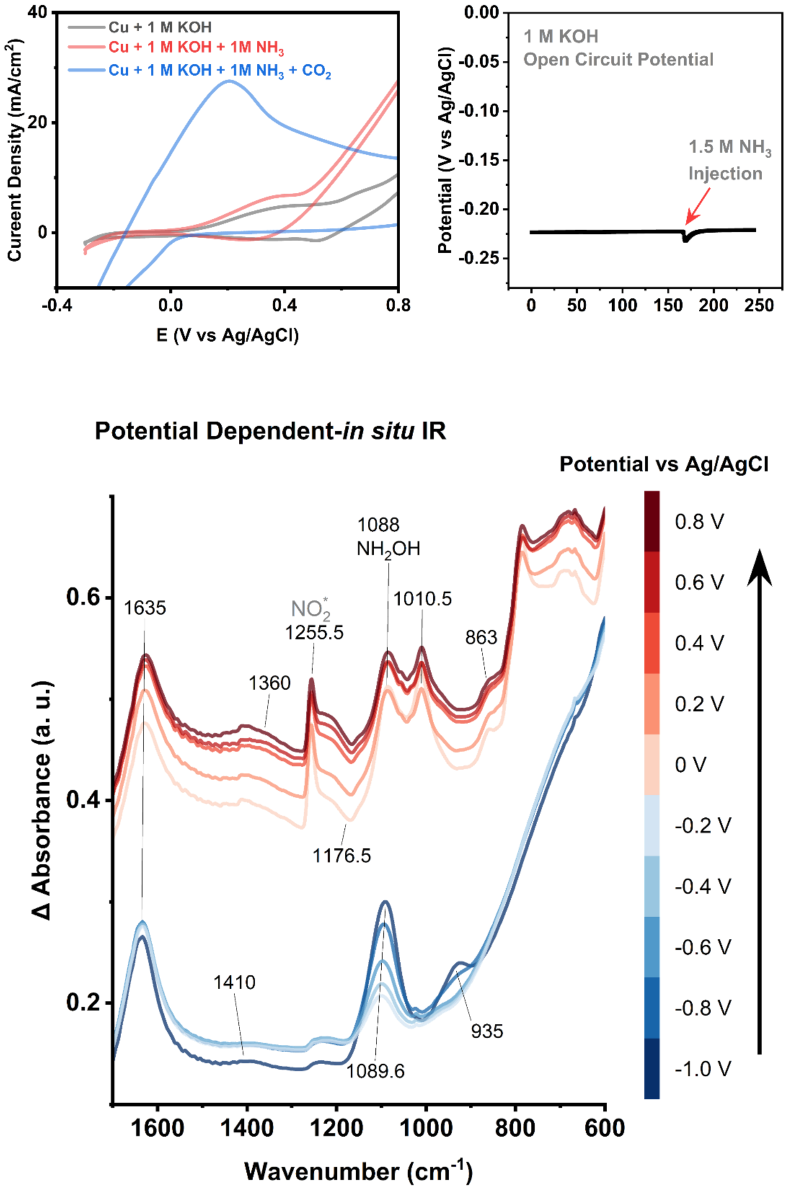

Figure S6. **IR spectroelectrochemical testing**. CVs of the Cu showing the oxidation of Cu, NH_3 and CO_2R intermediates. Spectra recorded as a function of applied potential from (-1.0 V to 0.8 V) in the condition of 1 M KOH, 1.5M NH_3 (b).

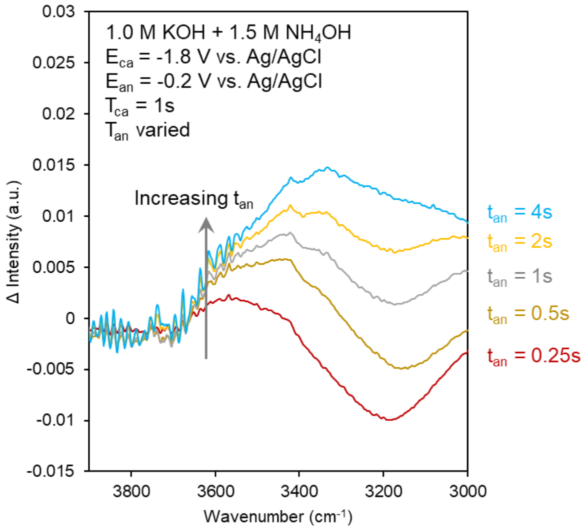

Figure S7. **IR under differential pulsing times.** Under a typical set of pulsing conditions in the absence of CO_2, the tentatively assigned N-H stretches from $*NH_2$ increase in intensity as the anodic pulse duration is increased from 0.25s to 4s.

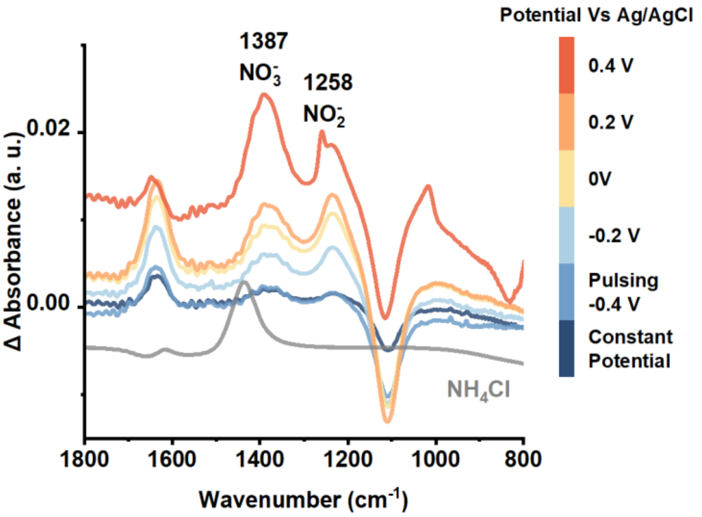

Figure S8. IR spectroelectrochemical testing. With the system at open circuit used as the background, spectra were acquired at static electrolysis (E_{ca} = -1.8V vs Ag/AgCl) and Pulsed electrolysis (E_{an} = - 0.4 V ~ 0.4 V) in the presence of NH_3 only.

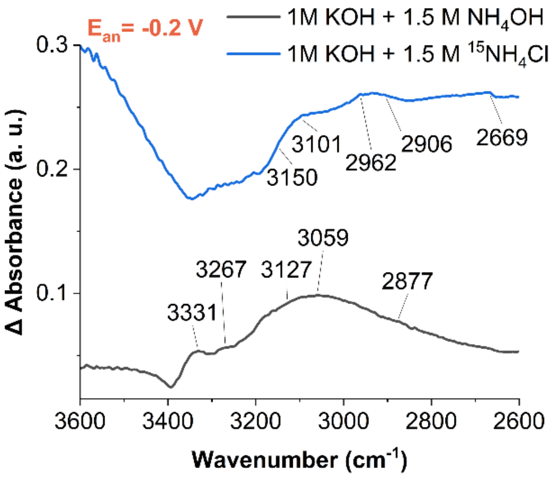

Figure S9. **Isotope studies**. $^{15}NH_4Cl$ was used to replace the NH_4OH to identify the IR bands associate the N-H bonds in the high-wavenumber region.

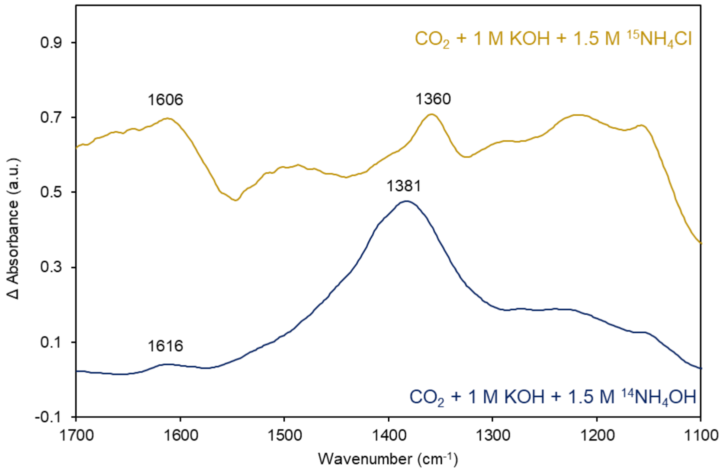

Figure S10. **Isotope labelling.** $^{15}NH_4Cl$ was used to identify potential vibrational modes associated with C-N products and intermediates in the low-wavenumber region. Bands that shift to lower frequencies are likely associated with $^{14}N/^{15}N$ -containing vibrational modes.

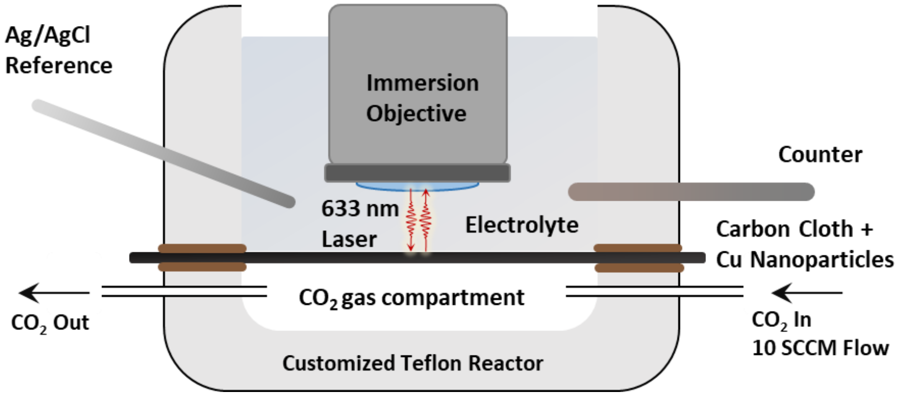

Figure S11. Raman measurement setup. Simplified schematic of the *in situ* Raman cell setup employed in this work. The cell was designed to mimic the reactor used for reactivity studies and similarly used a low volume and small distance between the objective and catalyst loaded electrode to maximize signal intensity.

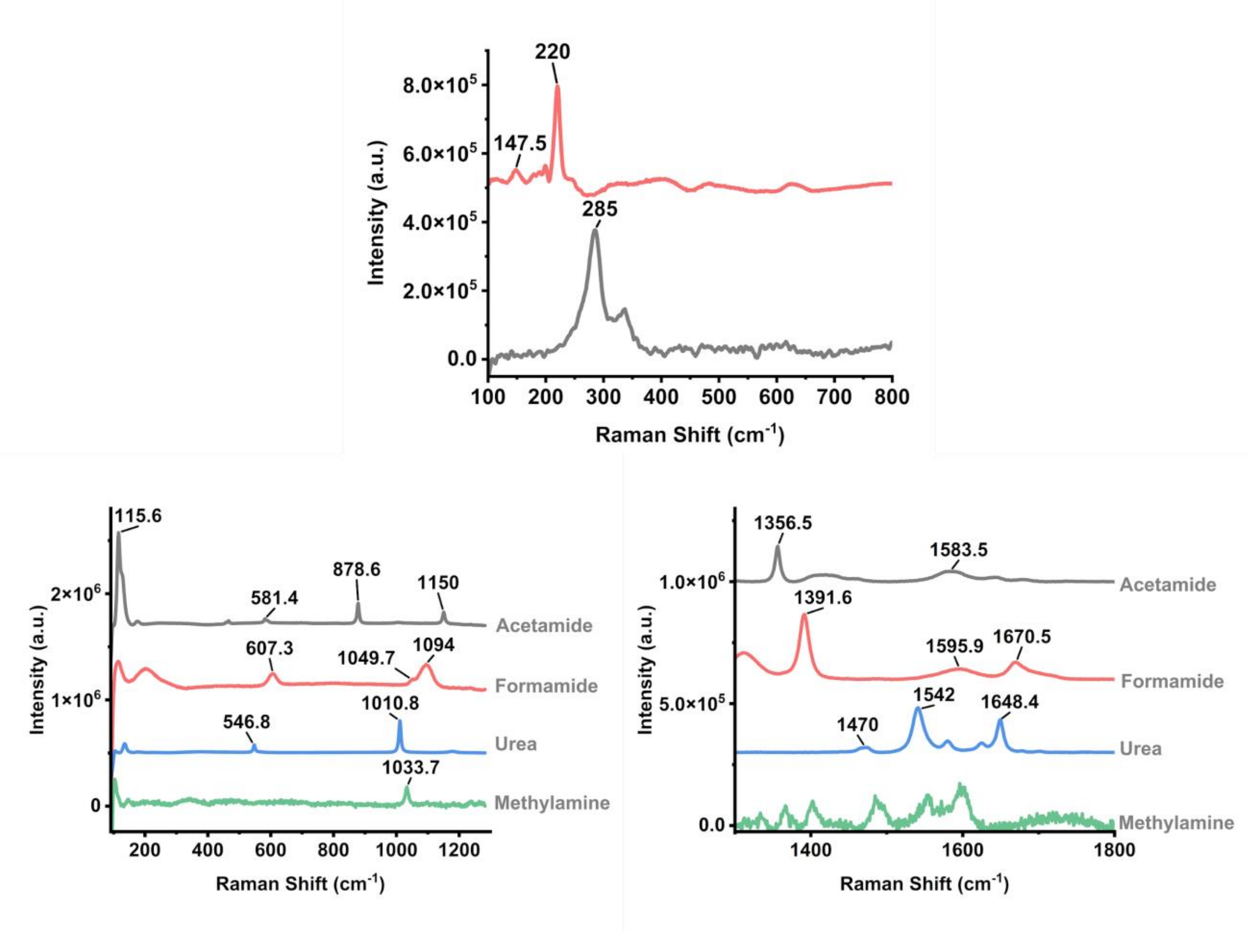

Figure S12. Raman Standards. Raman spectra of several reference compounds: Cu_2O and CuO (upper), C-N bond product (bottom). Spectra of each standard as either in a pure solid form, pure liquid form or highly concentrated solution were taken to maximize signal intensity.

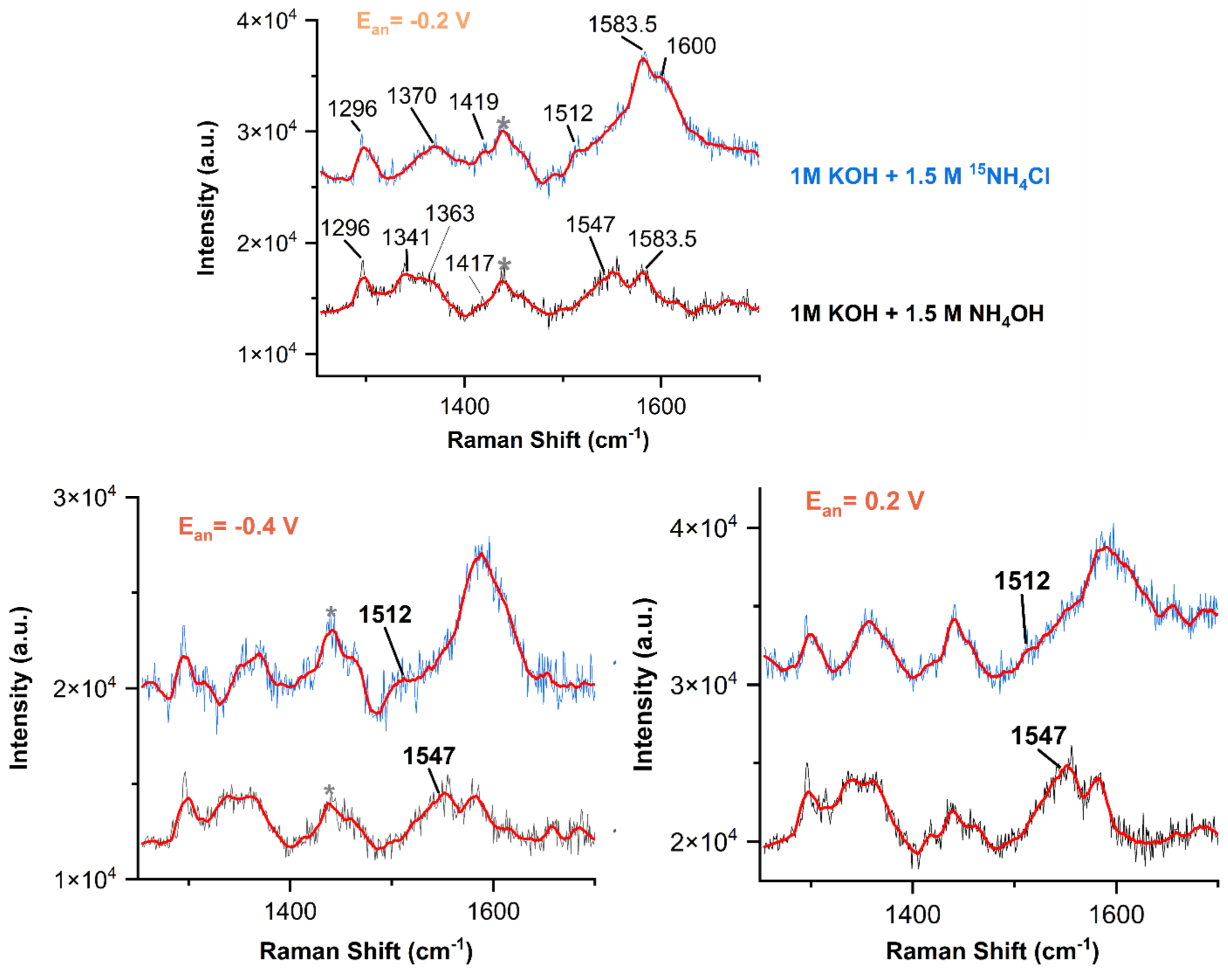

Figure S13. **Isotope Raman study.** We used ^{15}NH4Cl to replace the NH_4OH as the nitrogen source. The peak at 1547 cm^{-1} could be originating from the C-N bond, as it shifts to 1512 cm^{-1} when ^{15}N is used. We conduct the pulsed electrolysis in varied E_{an}, and the peak shift is same in each case.

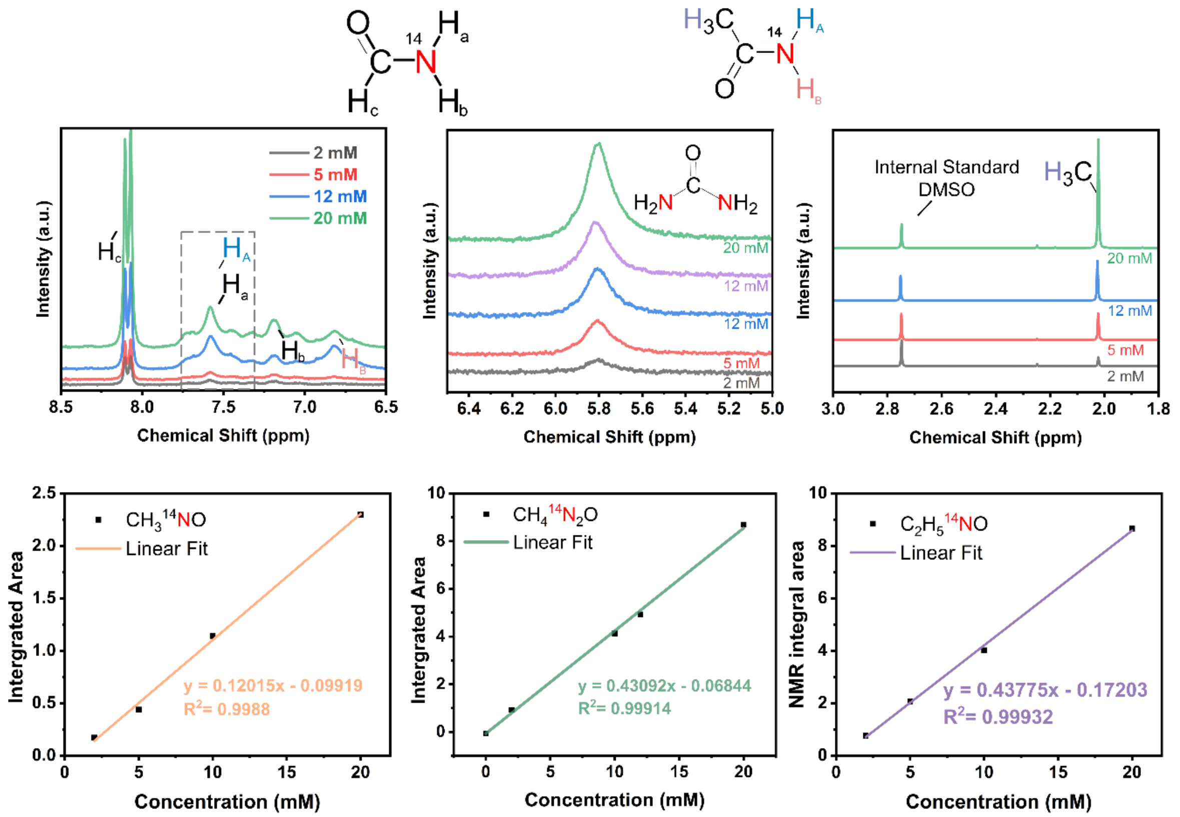

Figure S14. C-N bond product quantification. ^{1}H NMR analysis of NH_4Cl standard solutions (up) and calibration curve (bottom) of formamide, urea, and acetamide, respectively. The concentration of C-N bond products exhibits a linear relationship with the integral area of the characteristic peaks.

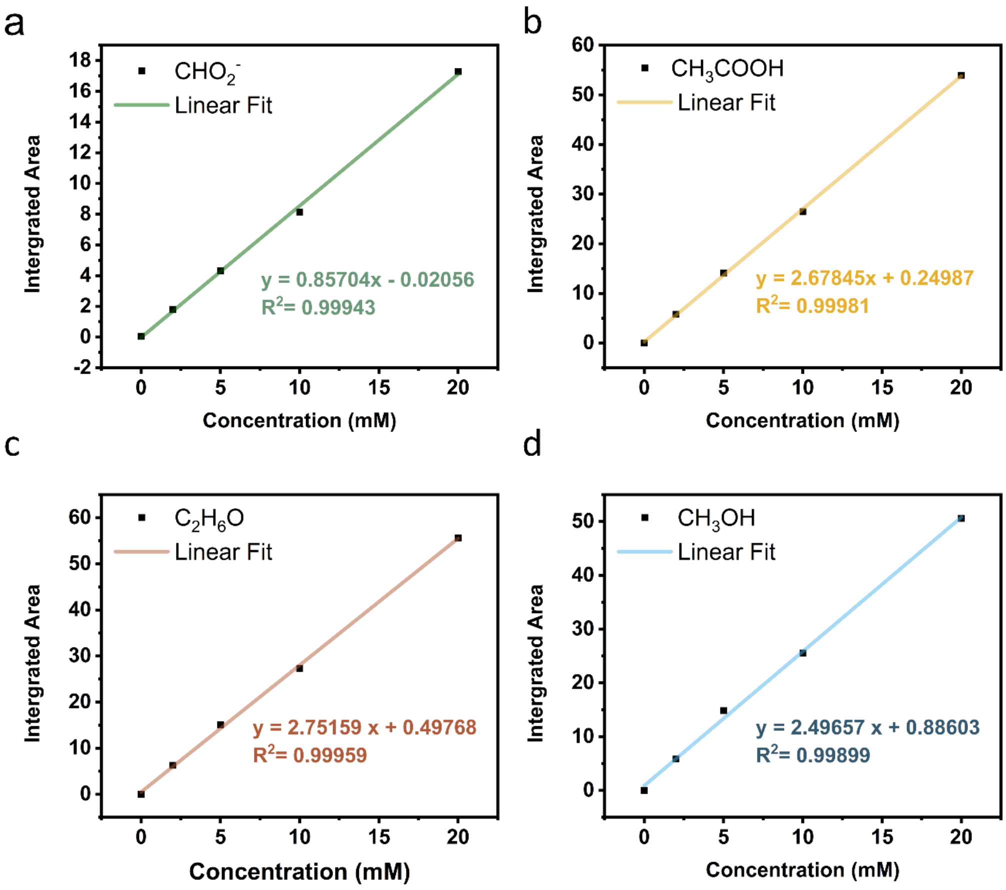

Figure S15. CO_2R product quantification. The calibration curve of formate, acetic acid, ethanol, and methanol respectively. The concentration of those bond products exhibits a linear relationship with the integral area of the characteristic peaks. The concentration ranges of formate (a), acetate (b), ethanol (c) and methanol (d) covered the concentrations observed in this work.

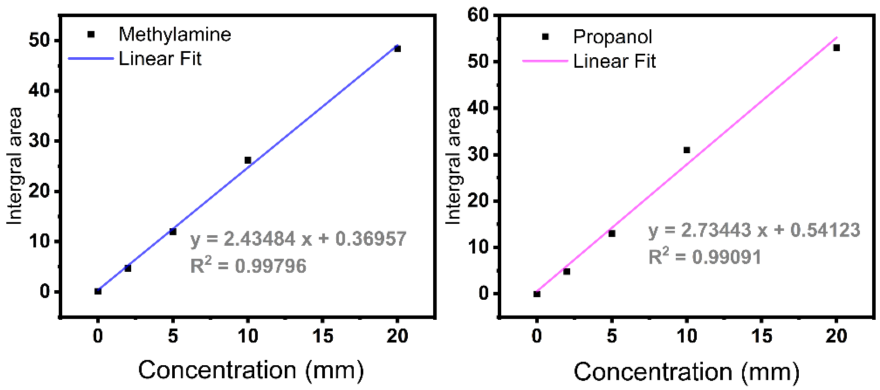

Figure S16. **Additional calibration curves**. The calibration curve of Methylamine and Propanol. The concentration of those products exhibits a linear relationship with the integral area of the characteristic peaks.

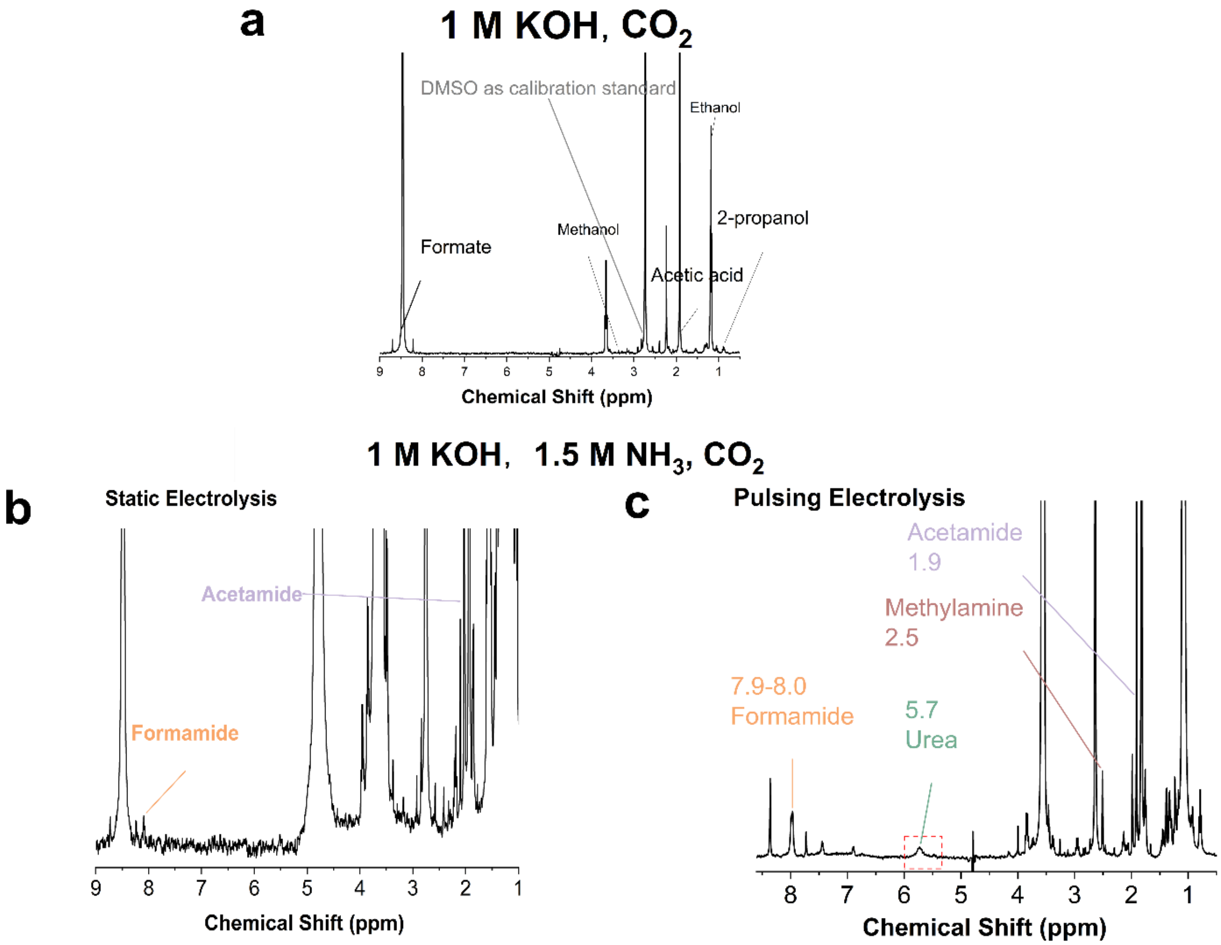

Figure S17. **NMR results**. NMR spectra of electrolyte of (a) without NH_3 after pulsing electrolysis, (b) with 1.5 M NH_3 after static electrolysis and (c) with 1.5 M NH_3 after pulsed electrolysis. No C-N bond product observed in a. No urea and methylamine could be detected in spectra b. there are four C-N bond related product could be detected in the spectra c. The signals with chemical shift between 7.8- 6.8 belong to the proton shift of N-H bond of C-N products. The pulsed electrolysis condition is 1s pulse at E_{an} = -0.2 V was followed 1s pulse at E_{ca} = -1.8 V and the loop was repeated for 30 min.

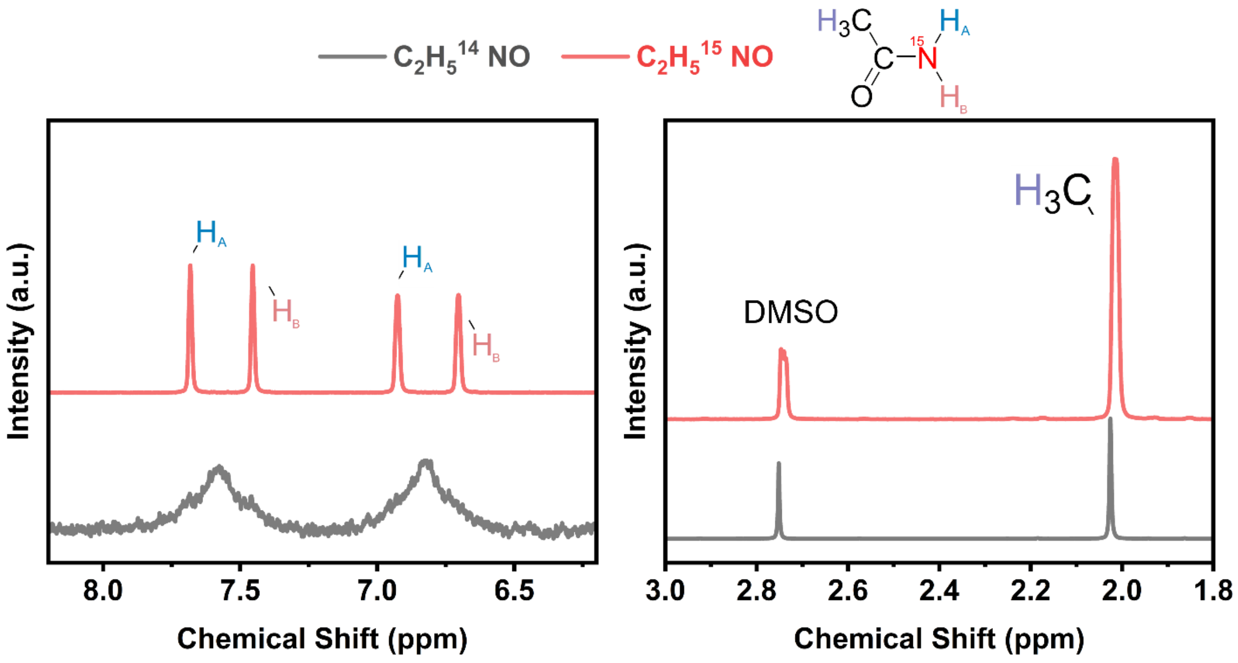

Figure S18. **Isotope labelling**. Standard NMR spectra of ^{14}N-acetamide (gray) and ^{15}N-acetamide (red). A clear sharp doublet can be observed for the N-H protons when ^{15}N is used while they are broader and singlets when ^{14}N is used.

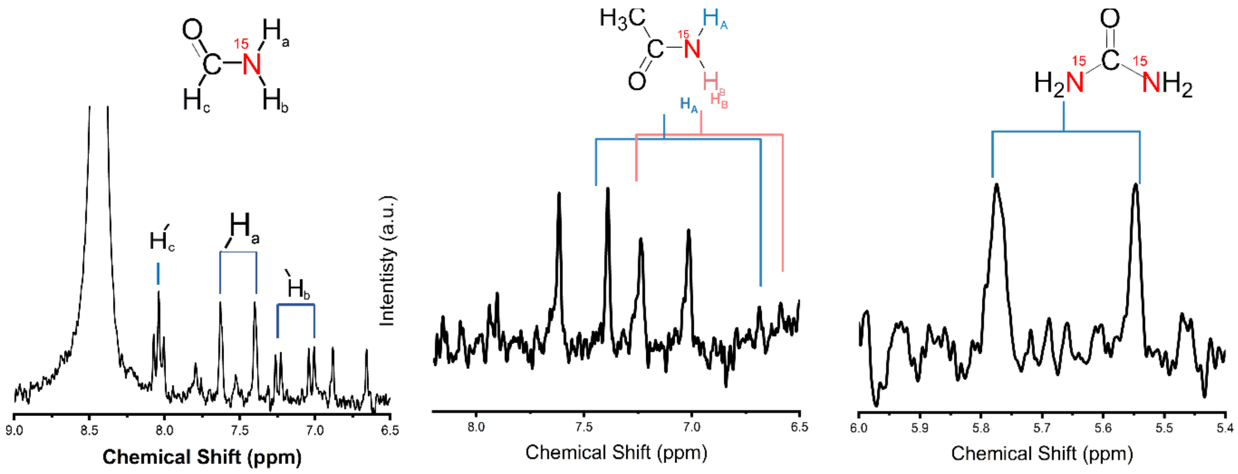

Figure S19. Isotope Labelling. NMR spectra of electrolyte solution after using $^{15}NH_3$ to replace the $^{14}NH_3$ to produce C-N bond products. The pulsed electrolysis condition is 1s pulse at E_{an} = -0.2 V was followed 1s pulse at E_{ca}=-1.8 V and the loop was repeated for 30 min.

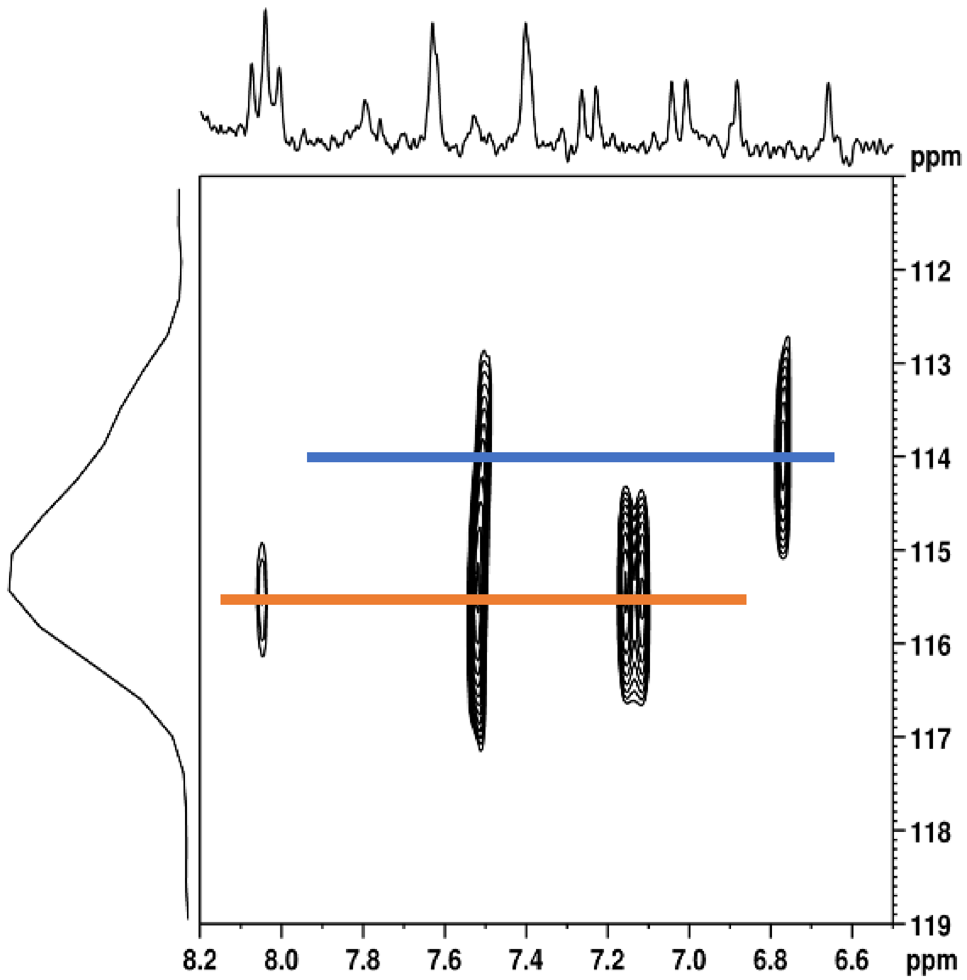

Figure S20. **2D NMR.** 1H-^{15}N heteronuclear single-quantum correlation (HSQC) to prove the formation of both acetamide and formamide. Cross-peaks are observed for the two chemically distinct amide hydrogens of acetamide (blue line d_N = 114.0 ppm). For formamide cross-peaks are observed from the two chemically distinct amide hydrogens as well as the hydrogen bound to the carbonyl (orange line d_N = 115.5 ppm). The pulsed electrolysis condition is 1s pulse at E_{an} = -0.2 V was followed 1s pulse at E_{ca}= -1.8 V and the loop was repeated for 30 min.

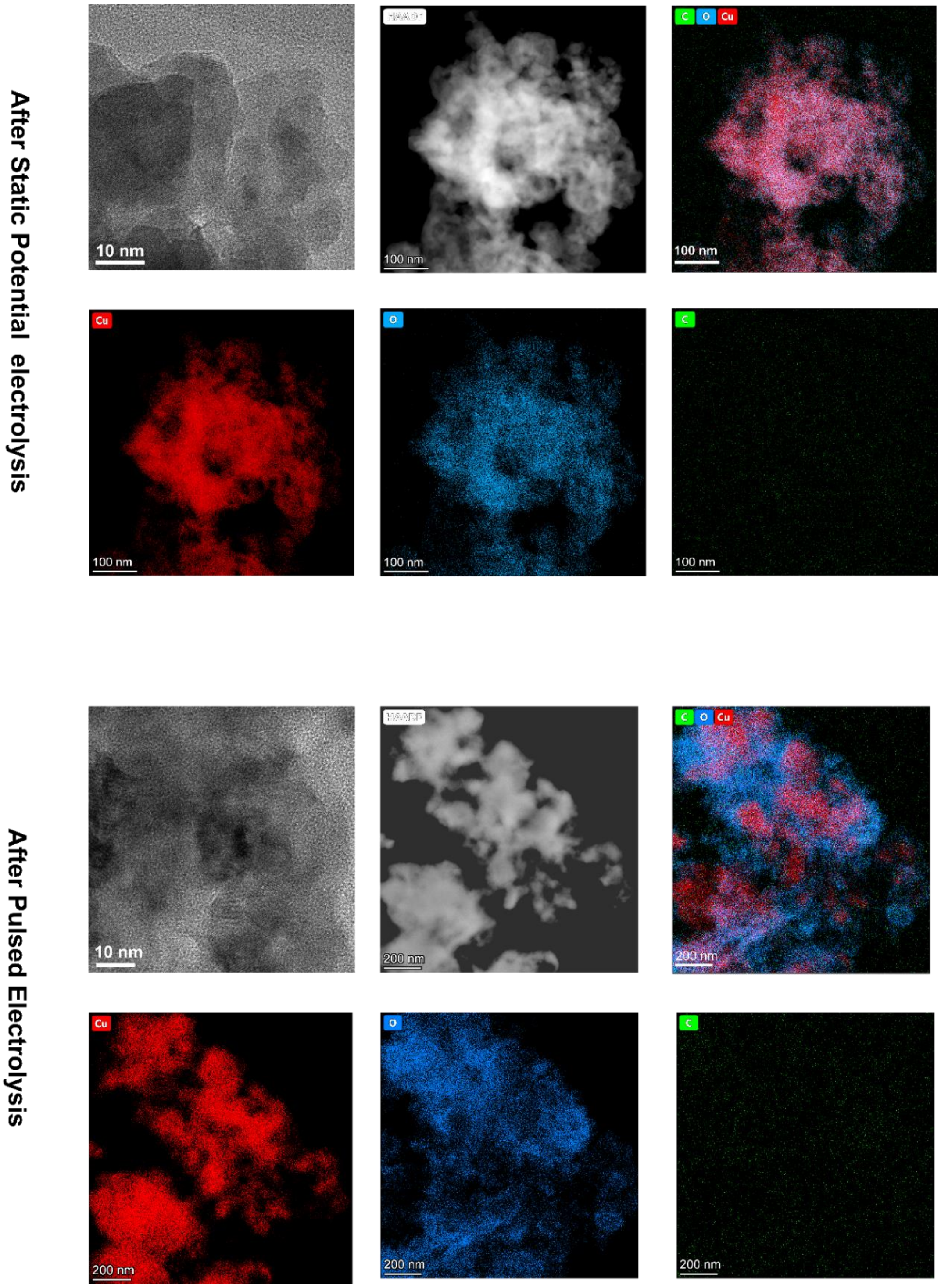

Figure S21. **TEM analysis.** High resolution TEM and high angle annular dark field image (HADDF) and EDS map of the catalyst particles after steady state and pulsed electrolysis. the electrolyte for both static and pulsed electrolysis is same: 1 M KOH and 1.5 M NH_4OH. For static electrolysis, the electrode applied a chronoamperometry at -1.8 V vs Ag/AgCl for 30 mins. For pulsed electrolysis, the electrode applied a cathodic potential are E_{ca} = 1.8 V for 1s then E_{an} = -0.2 V for 1s. In total, the pulsed condition is conducted 30 min.

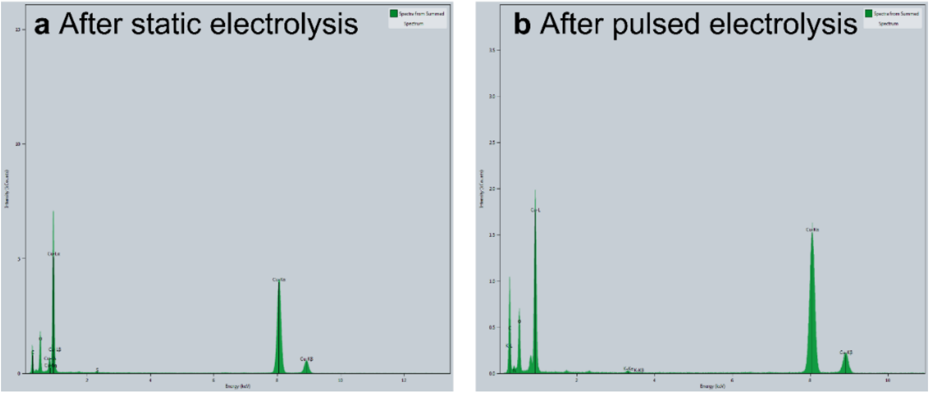

Figure S22. EDS Analysis. EDS spectra of the catalyst after static electrolysis (a) and after pulsed electrolysis (b). The electrolysis condition same with **Figure S19**.

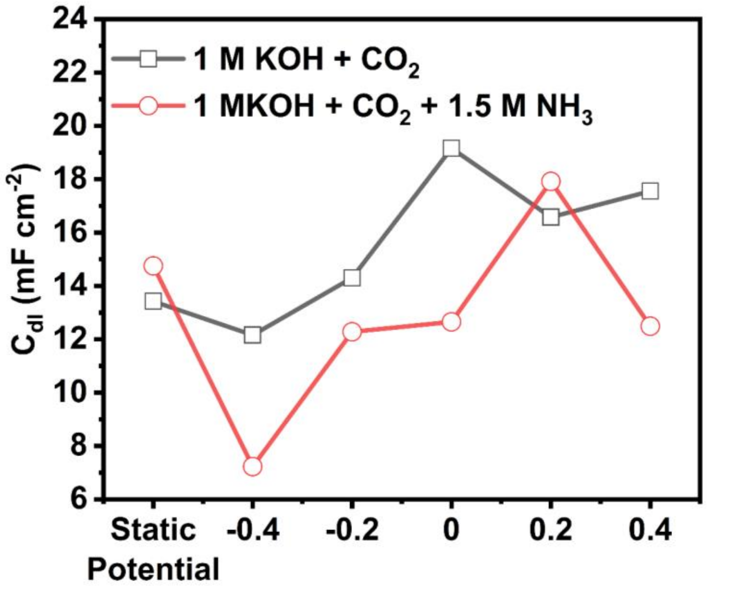

Figure S23. **Surface area measurements.** Double-layer capacitance as determined by cyclic voltammetry. The electrochemical surface area after 0.5 h electrolysis was obtained by double layer capacitance with cyclic voltammetry (CV) in the non-Faradaic region with variable scan rates. CVs were acquired out between -0.2 to 0 V versus Ag/AgCl, with scan rate of 5, 20, 40, 60, 80, 100 and 120 mV s^{-1}.

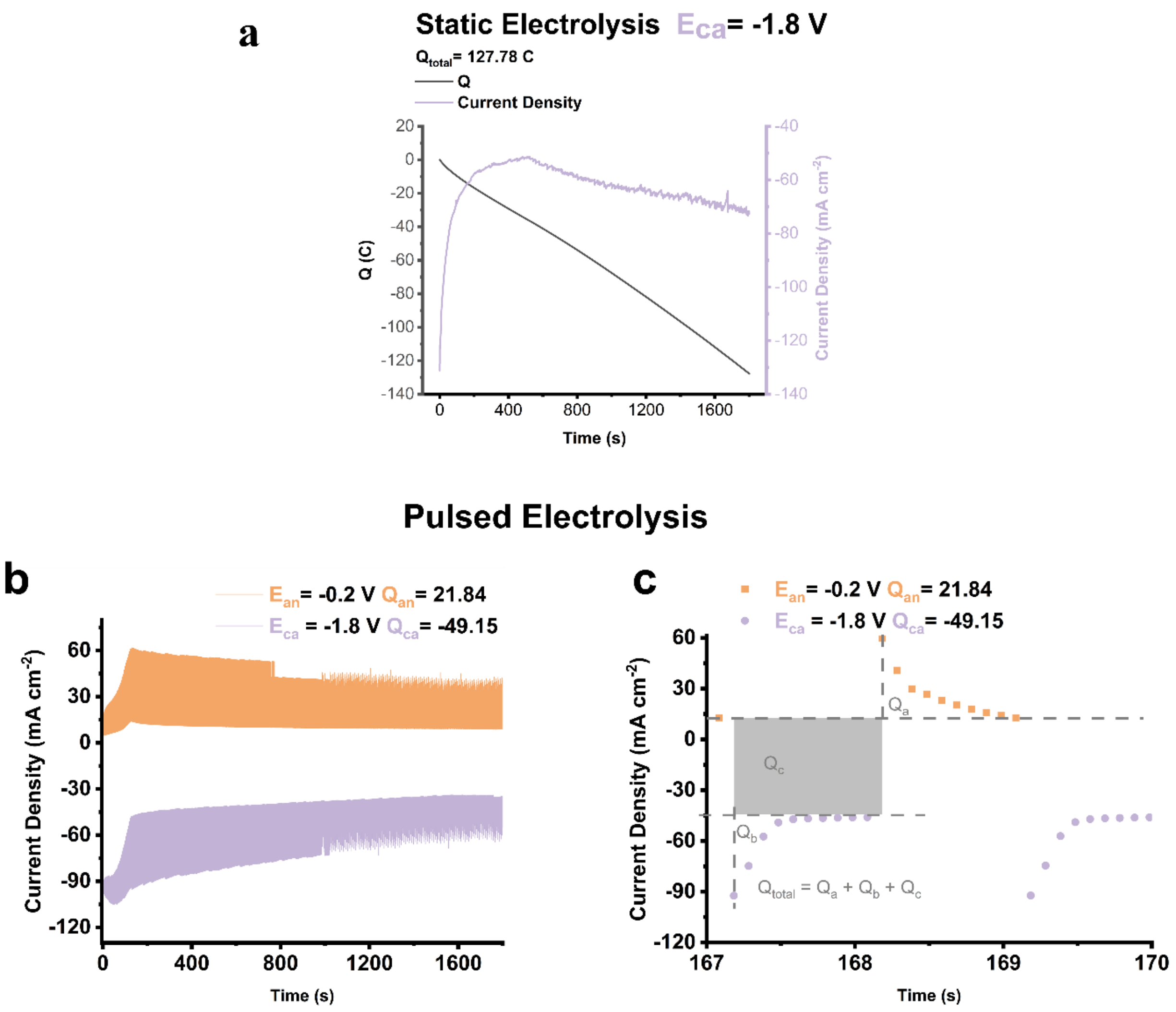

Figure S24. Electrochemical traces. Typical current density vs. time trace for both a steady electrolysis (a) and pulsed electrolysis (b) and zoom-in (c). The listed Q is a representative amount shown for reference. Example (c) of a current transient of pulsed electrolysis at t_{ca} = 1s and t_{an} = 1s with the corresponding oxidative (Q_a), reductive (Q_b) and total charge passed through the circuit during electrolysis ($Q_{total} = Q_a + Q_b + Q_c$).

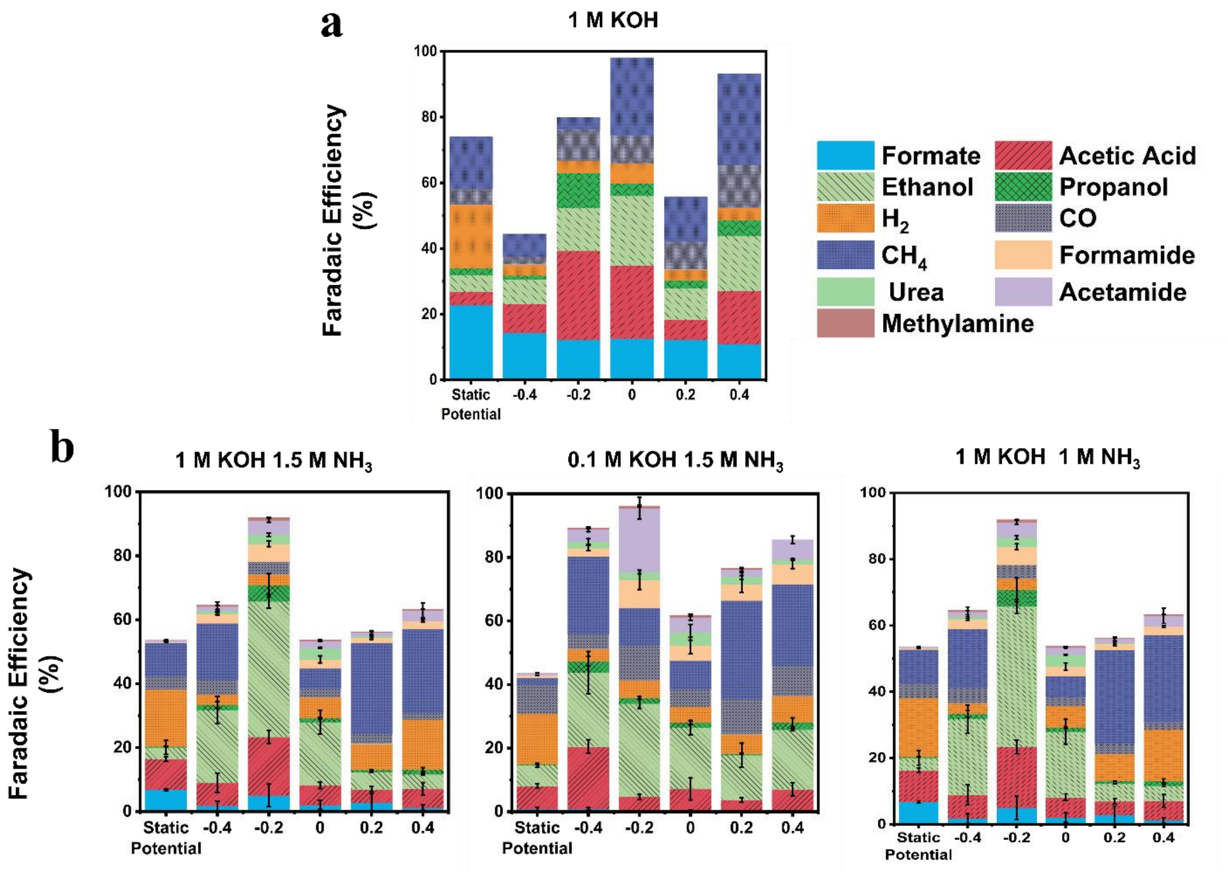

Figure S25. **Total Faradaic Efficiencies**. Faradaic efficiencies for the static electrolysis and pulsed electrolysis in the absence of NH3 (a) and (b) with NH_3. For static electrolysis, the potential sets as -1.8 V. For pulsed electrolysis, the values for the cathodic time and anodic time are t_{ca} = 1s and t_{an} = 1s, and the cathodic potential are E_{ca} = 1.8 V and E_{an} = -0.4 V to 0.4 V, respectively. Each system was fed with CO_2 at a constant flow (10 sccm). The results in line with the results of the ECSA detection, which means the selectivity towards CH_4 has some relation with the surface roughness[15,21]. The highest Faradaic efficiency of CH_4 is 0 V in the absence of NH_3 and 0.2 V in the presence of the NH_3. Each electrolysis conditions were repeated for more than 3 times to determine standard deviations between measurements.

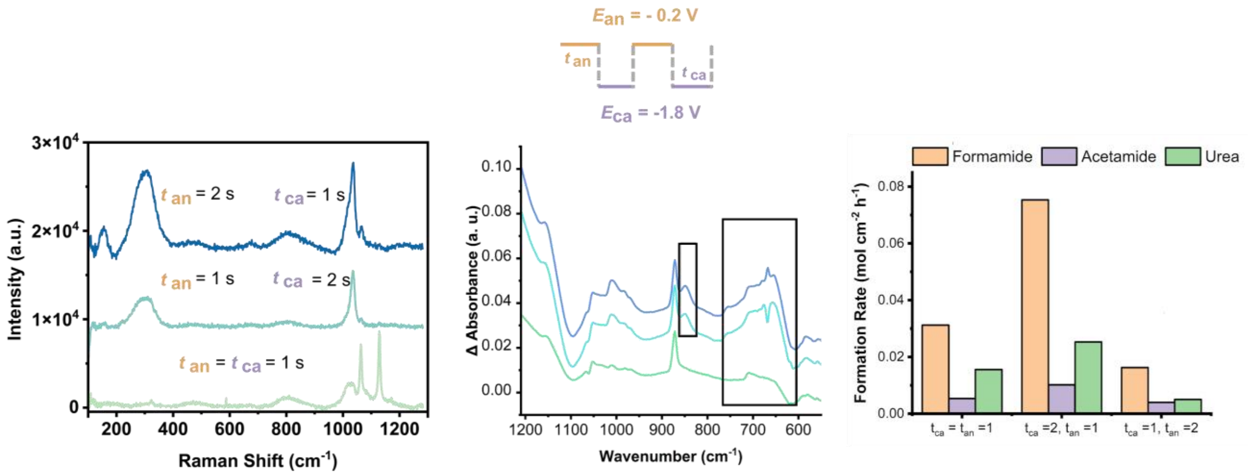

Figure S26. Effects of the durations of the anodic (t_{an}) and cathodic (t_{ca}) pulses. From left to right, the graph corresponds to the *in situ* Raman Spectra, the *in situ* IR spectra and the formation rates. Doubling the anodic potential would promote the formation of oxygenated copper species, as indicated by the Raman spectra. Formation rates of formamide doubled when increasing the cathodic pulses time, and the formation rate decreases when doubling the anodic pulse duration.

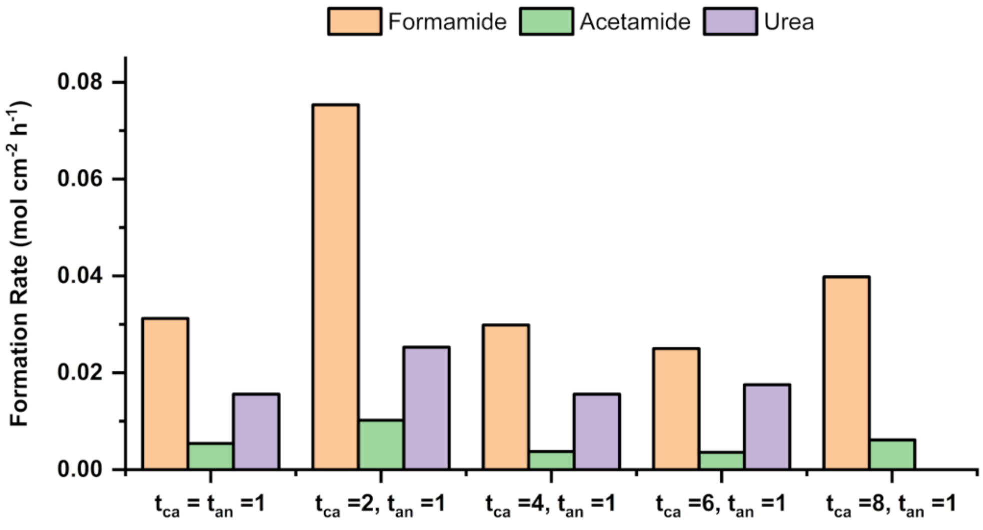

Figure S27. **Effect of the durations of the cathodic (t_{ca}) pulses times on product formation rates.** The reaction conditions entailed 1.5 NH_3 and 1.0 M KOH, E_{ca} = -1.8 V and E_{an} = -0.2 V vs. Ag/AgCl.

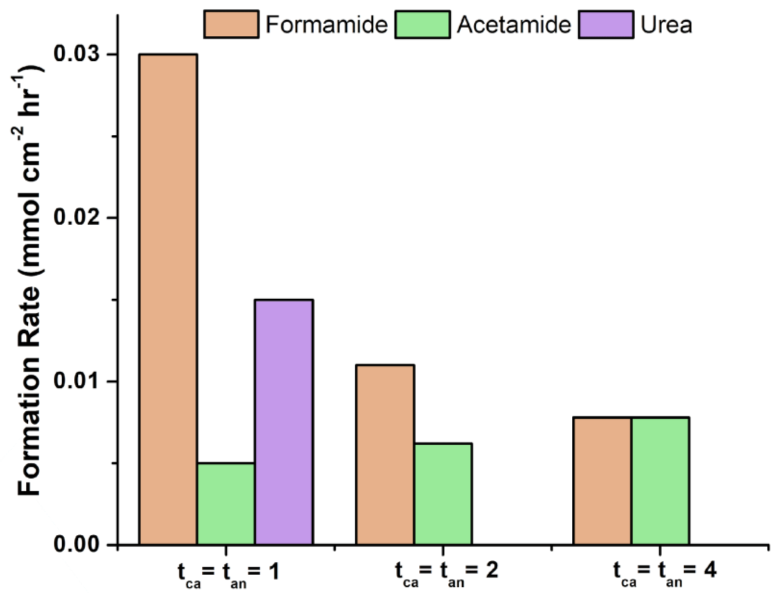

Figure S28. **Effect of total pulse durations (1s, 2s or 3s) on product formation rates**. The reaction conditions were 1.5 NH_3 and 1.0 M KOH, E_{ca} = -1.8 V and E_{an} = -0.2 V vs. Ag/AgCl.

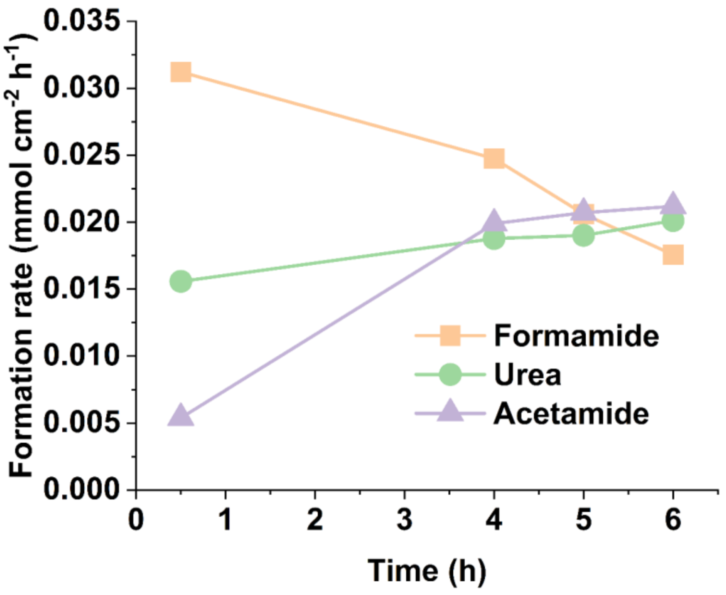

Figure S29. **Extended Electrolysis**. Long term operation was tested at E_{an} = -0.2 V for 1s and E_{ca} = -1.8 V vs Ag/AgCl for 1s. The formation rate of formamide is decreasing, while the acetamide and urea formation rate is increasing might attribute to the interplay of the gradual consumption of the ammonia and the drop of the electrolyte pH when operating.

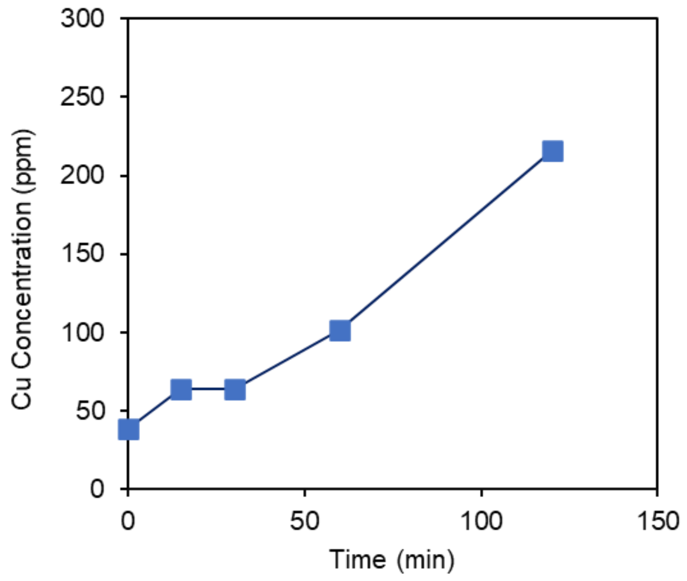

Figure S30. **Cu dissolution analysis.** Cu was measured in the electrolyte (E_{an} = -0.2 V for 1s and E_{ca} = -1.8 V vs Ag/AgCl for 1s.) as a function of time with ICO-OES measurements and the data shows a continual accumulation of Cu in the electrolyte over time.

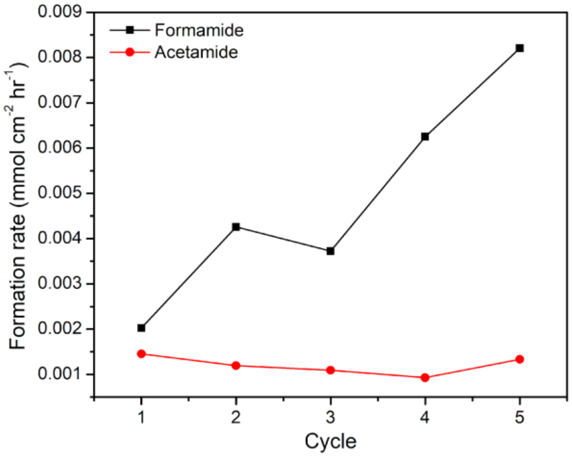

Figure S31. **Extended time analysis**. The production rates of C-N products over multiple 1-hr cycles in which the electrolyte was changed and electrode rinsed off. Changes is formation rates indicate possible changes to the catalyst over time, likely caused by Cu dissolution and redeposition. The reaction conditions were 1.5 NH_3 and 1.0 M KOH, E_{ca} = -1.8 V and E_{an} = -0.2 V vs. Ag/AgCl.

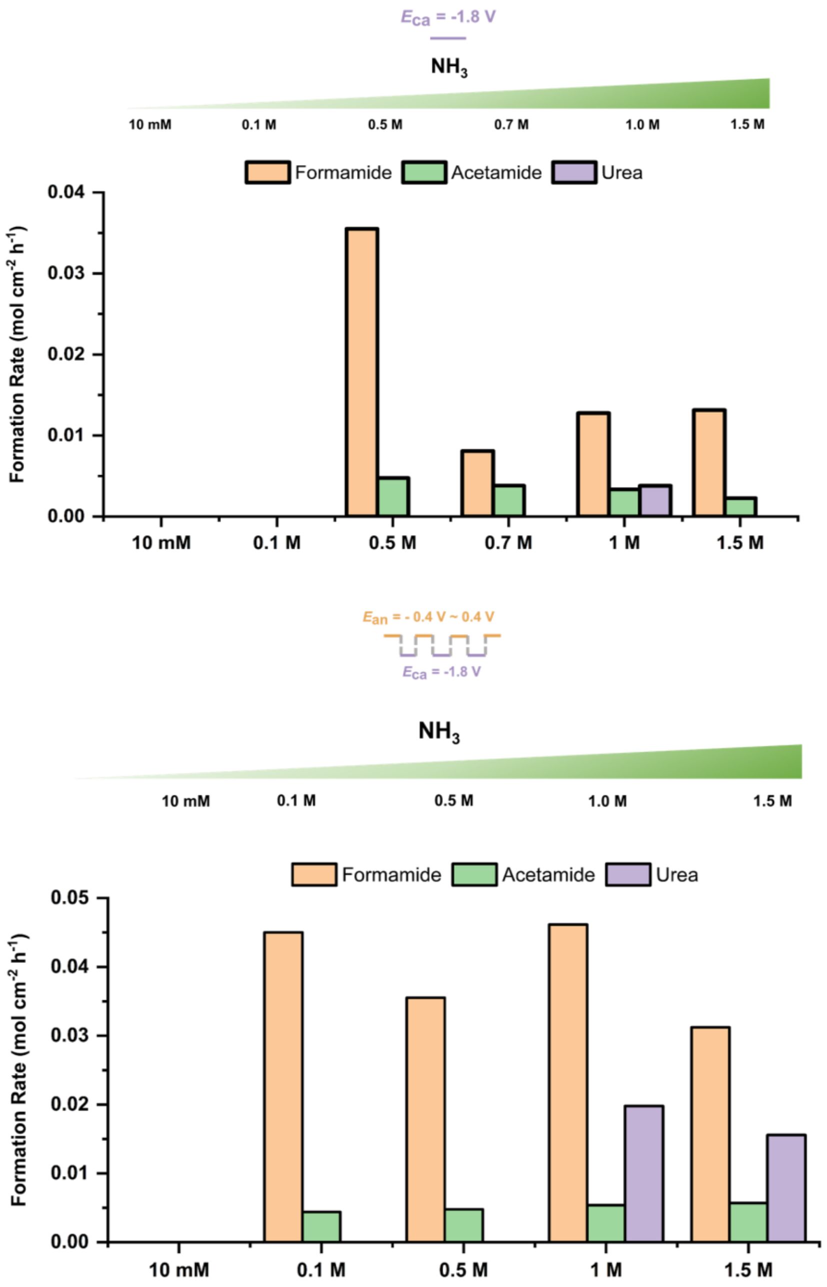

Figure S32. **Effects of concentration of NH_3.** The reaction conditions entailed 1.5 NH_3 and 1.0 M KOH, E_{ca} = -1.8 V and E_{an} = -0.2 V vs. Ag/AgCl. The trends for formamide, acetamide and urea follow differing trends whether pulsed voltage is used or not.

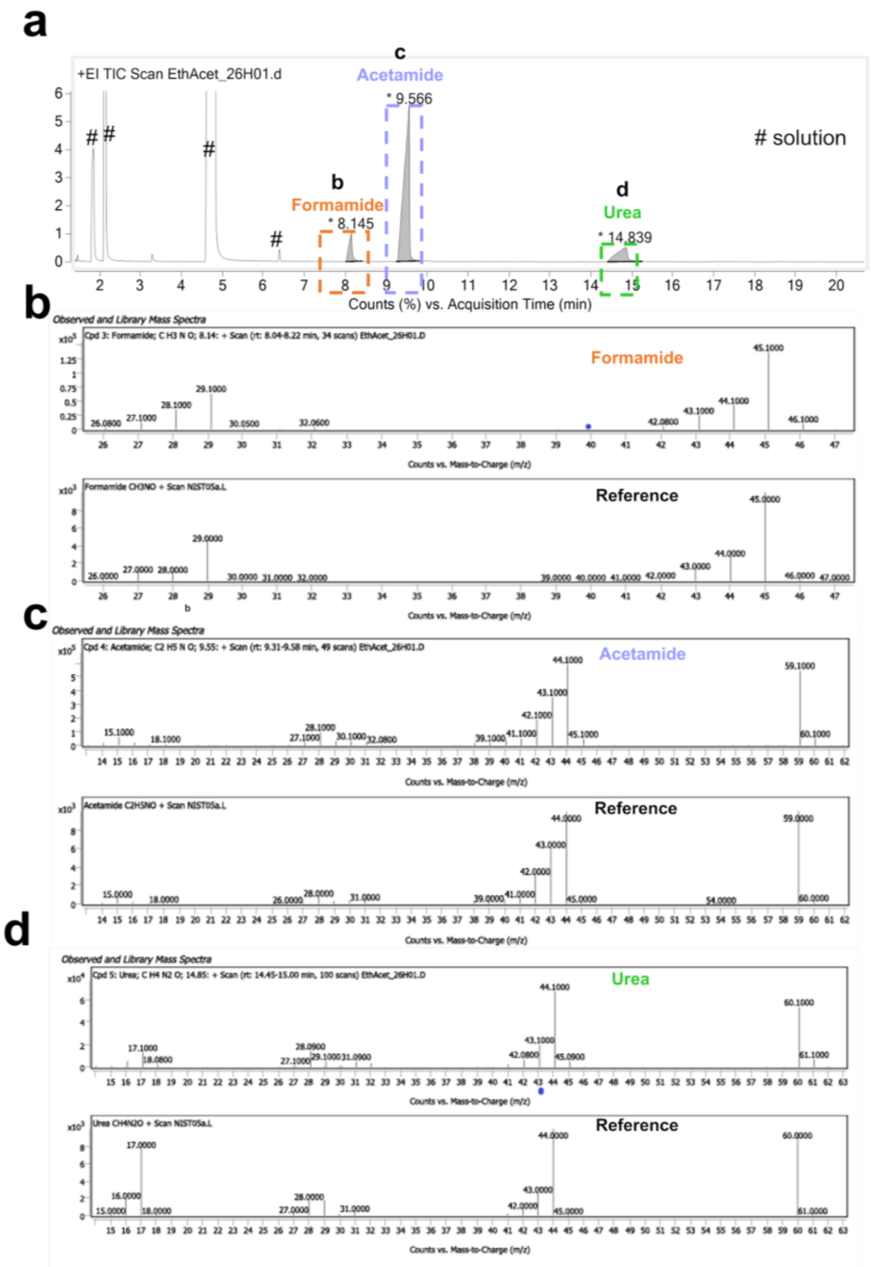

Figure S33. **Qualitative analysis of C-N bond formation by GC-MS**. Beside solvent related peaks, formamide, acetamide and urea were identified in the sample (a) with matching scores of 98.13 (b), 98.64 (c) and 96.11 (d) respectively.

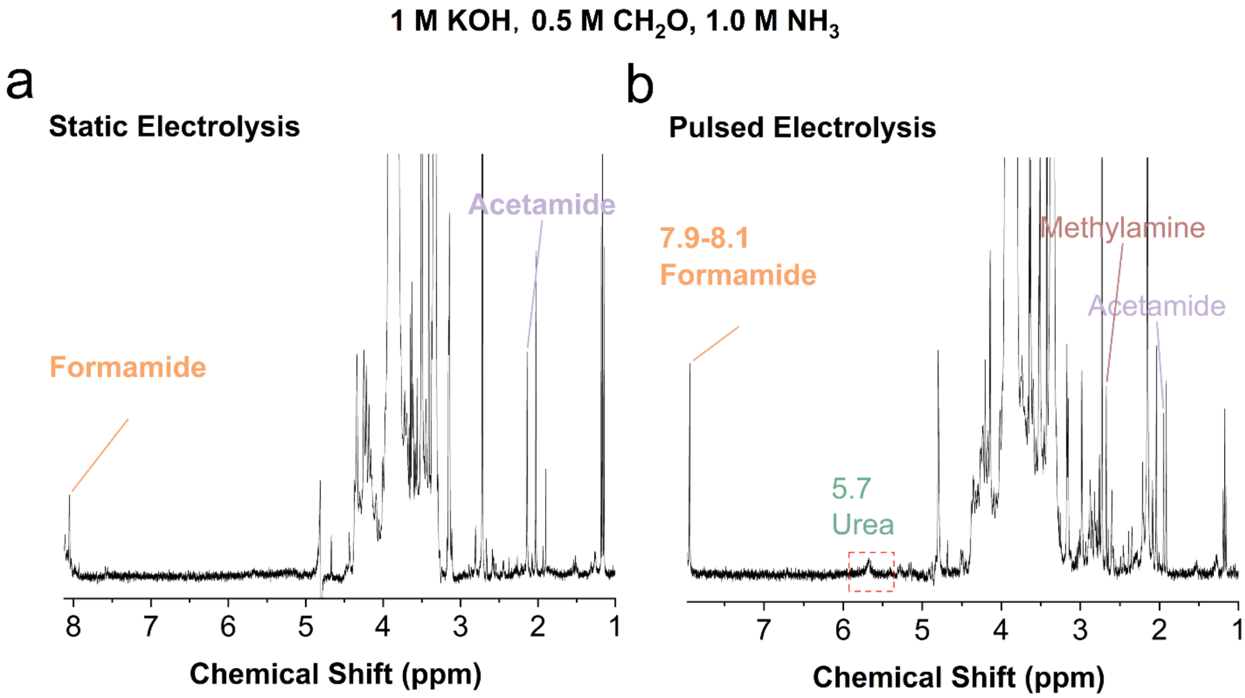

Figure S34. NMR Analysis. NMR spectra of 0.5 M CH_2O used as the C-source to replace CO_2. The scale of NMR spectra of static electrolysis and pulsing electrolysis remains the same to compare the change in product concentration. When static electrolysis was used (a) urea was not detected and the rest of the C-N products were much harder to detect as compared to the case when pulsed electrolysis was used (b).

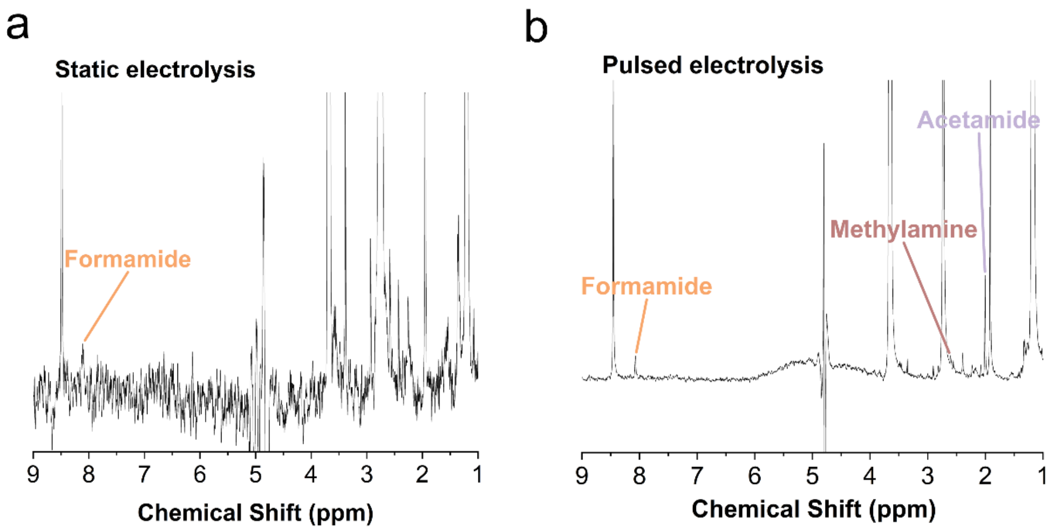

Figure S35. NMR Comparison. NMR spectra of 0.5 M NO_3^- as N-source to replace NH_4OH. Only Formamide can be observed in the static electrolysis condition (a), while both formamide, acetamide and methylamine are present in the electrolysis solution after pulsed electrolysis (b).

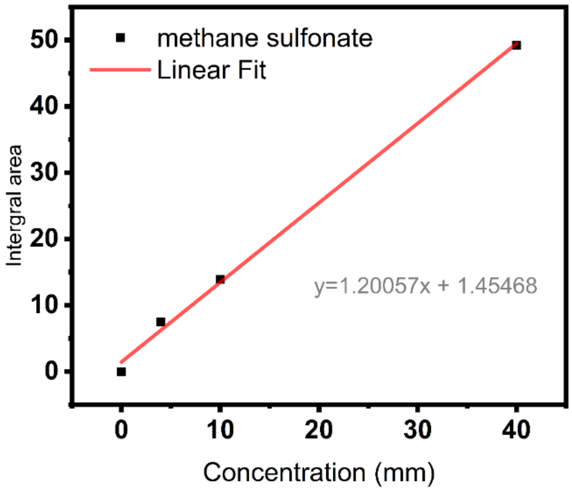

Figure S36. **MS quantification**. The calibration curve of methane sulfonate obtained from different concentrations of standard methane sulfonate solutions in the employed 1.0 M KOH electrolyte using DMSO as an internal standard.

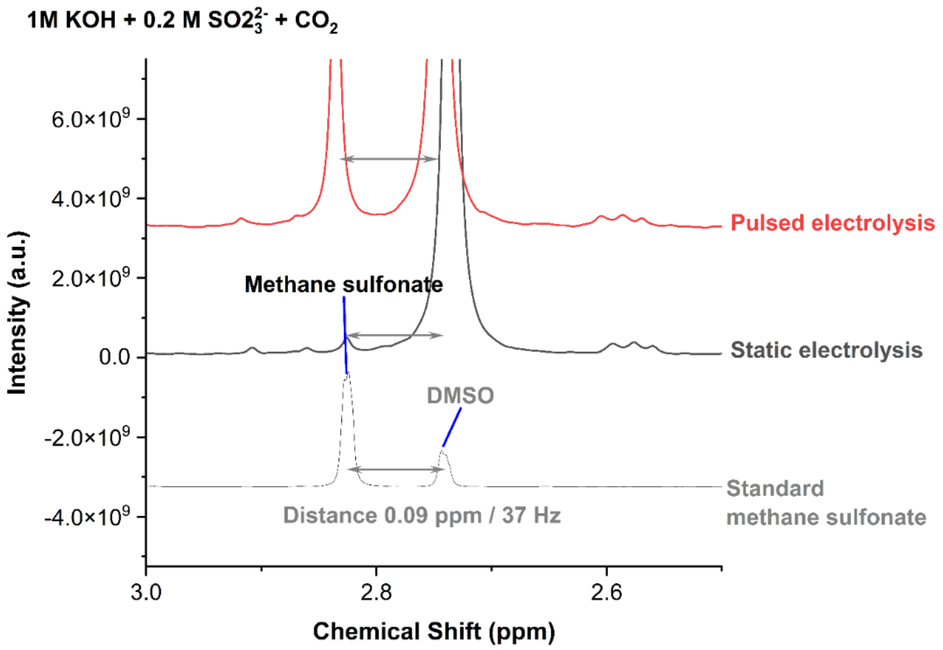

Figure S37. **MS analysis**. NMR spectra of standard methane sulfonate, static electrolysis, and pulsing electrolysis (from bottom to top). The scale of NMR spectra of static electrolysis and pulsing electrolysis remains the same to illustrate the change in product concentration. The chemical shift is a bit of different likely due to the pH being different in these two electrolysis conditions. However, the distance of chemical shift between DMSO and methane sulfonate remains the same.

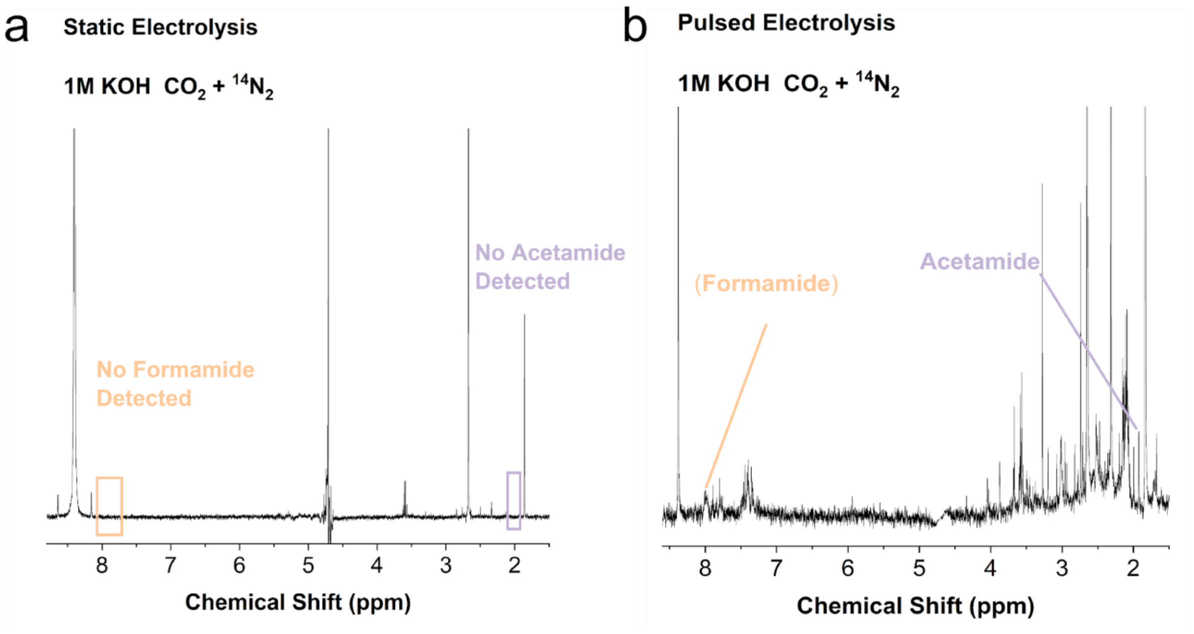

Figure S38. Expanding Scope. NMR spectra of co-electrolysis CO_2 and N_2 under the static electrolysis case (a) and pulsed electrolysis case (b). The calculated Faradaic efficiency for formamide and acetamide is 0.98 % and 0.58 %. It should be noted that the current Faradaic efficiency is not high enough to prove the N-source is directly from N_2.

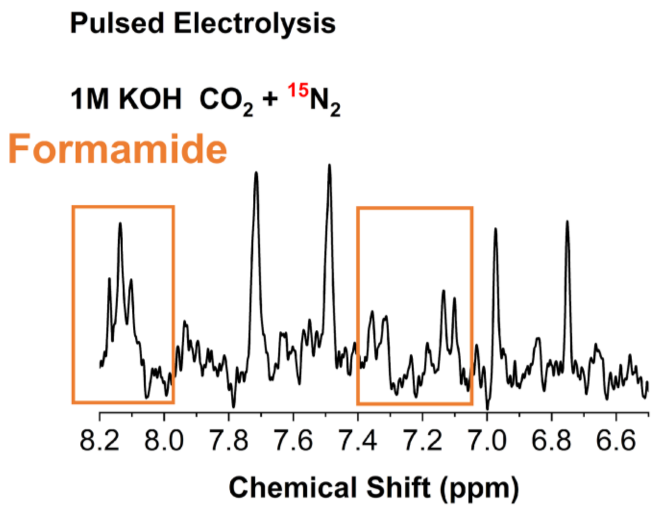

Figure S39. Isotope labelling. $^{15}N_2$ Isotope experiment was conducted to verify the formation of formamide and acetamide. $^{15}N_2$ flowed into the GDE cell for 1h.

Table S3. Summary of C-N bond product formation. Note: the total time (cathodic plus anodic) was used to calculate formation rates and partial current densities. Urea production was assigned 2e^-, even though it is electrochemically neutral (0e^-) so it can be roughly compared with the FE and I of other products.

Electrolyte	Reactants	Ecal (vs. Ag/AgCl)	Ean (vs. Ag/AgCl)	Product	FE (%)	F.R. (mmols*hr-1*cm-2)	I (mA/cm2)
1.0 M KOH, 1.5 M NH3	CO2, NH3	-1.8 V	None	Formamide	0.38	0.013	0.70
				Urea	0	0	0.00
				Acetamide	0.53	0.0023	0.49
	CO2, NH3	-1.8 V	-0.4 V	Formamide	2.85	0.034	1.82
				Urea	0.95	0.014	0.75
				Acetamide	1.4	0.028	6.00
	CO2, NH3	-1.8 V	-0.2 V	Formamide	5.45	0.031	1.66
				Urea	2.79	0.016	0.86
				Acetamide	4.56	0.0054	1.16
	CO2, NH3	-1.8 V	0 V	Formamide	2.84	0.015	0.80
				Urea	3.61	0.031	1.66
				Acetamide	2.11	0.0045	0.96
	CO2, NH3	-1.8 V	0.2 V	Formamide	1.73	0.017	0.91
				Urea	0.38	0.0054	0.29
				Acetamide	1.2	0.0031	0.66
	CO2, NH3	-1.8 V	0.4 V	Formamide	2.48	0.013	0.70
				Urea	0	0	0.00
				Acetamide	3.27	0.0027	0.58
0.1 M KOH, 1.5 M NH3	CO2, NH3	-1.8 V	None	Formamide	0.76	0.019	1.02
				Urea	0	0	0.00
				Acetamide	0.75	0.0036	0.77
	CO2, NH3	-1.8 V	-0.4 V	Formamide	2.62	0.031	1.66
				Urea	2.06	0.015	0.80
				Acetamide	3.84	0.016	3.43
	CO2, NH3	-1.8 V	-0.2 V	Formamide	8.73	0.062	3.32
				Urea	2.49	0.025	1.34
				Acetamide	20.03	0.023	4.93
	CO2, NH3	-1.8 V	0 V	Formamide	4.75	0.023	1.23
				Urea	4.15	0.012	0.64
				Acetamide	4.78	0.011	2.36
	CO2, NH3	-1.8 V	0.2 V	Formamide	5.17	0.041	2.20
				Urea	2.51	0.021	1.13
				Acetamide	2.12	0.0045	0.96
	CO2, NH3	-1.8 V	0.4 V	Formamide	6.32	0.03	1.61
				Urea	1.54	0.021	1.13
				Acetamide	6.16	0.008	1.72
1.0 M KOH, 1.0 M NH3	CO2, NH3	-1.8 V	None	Formamide	0.54	0.013	0.70
				Urea	0.068	0.0038	0.20
				Acetamide	1.02	0.0033	0.71
	CO2, NH3	-1.8 V	-0.4 V	Formamide	3.52	0.031	1.66
				Urea	1.76	0.016	0.86
				Acetamide	3.71	0.0045	0.96
	CO2, NH3	-1.8 V	-0.2 V	Formamide	4.72	0.046	2.47
				Urea	1.98	0.02	1.07
				Acetamide	2.17	0.0047	1.01
	CO2, NH3	-1.8 V	0 V	Formamide	2.47	0.036	1.93
				Urea	0.68	0.0092	0.49
				Acetamide	4.23	0.008	1.72
	CO2, NH3	-1.8 V	0.2 V	Formamide	3.5	0.033	1.77
				Urea	2.21	0.0093	0.50
				Acetamide	6.71	0.012	2.57
	CO2, NH3	-1.8 V	0.4 V	Formamide	5.65	0.036	1.93
				Urea	1.33	0.0044	0.24
				Acetamide	5.32	0.0034	0.73

Table S4. Raman and IR Peak assignment.

**Band Position (cm^{-1})**	**Infrared/Raman (Figure)**	**Potential Assignment**
209-220	Raman (4b)	Cu_2O
320	Raman (4b)	$Cu\text{-}C_x$
518, 619	Raman (4b)	CuO_x
635, 675	Raman (4b)	Cu-OH
983	Raman (4b)	HCO_3^-
1018	Raman (4b)	
1033	Raman (4b)	HCO_3^-
1067	Raman (4b)	CO_3^{2-}
1129	Raman (4b)	
1341	Raman (4c)	HCO_3^-
1363	Raman (4c)	$*CO_2^-$
1454	Raman (4c)	COOH
1547	Raman (4c)	C-N
1584	Raman (4c)	$*CO_2^-$
3564-3059	Infrared (4d)	N-H
2887	Infrared (4d)	C-H
1616	Infrared (4e)	$C\text{-}N/HCO_3^-$
1639	Infrared (4e)	H_2O
1450	Infrared (4e)	
1389	Infrared (4e)	NO_3^-
1232	Infrared (4e)	NO_2^-
1244	Infrared (4e)	
1051	Infrared (4e)	
871	Infrared (4e)	

666	Infrared (4e)	Cu-OH

Table S5. Partial Current Densities and Faradaic Efficiencies that corresponds to Figure 6.

Reactant	**Static Electrolysis**			**Pulsed Electrolysis**		
	Formation Rate $mM*h^{-1}*cm^{-1}$	Current Density mA/cm^2	Faradaic Efficiency %	Formation Rate $mM*h^{-1}*cm^{-1}$	Current Density mA/cm^2	Faradaic Efficiency %
CH_2O NH_3	$HCONH_2$					
	0.011	1.9	1.57	0.041	2.66	2.61
	CH_3CONH_2					
	0.013	9.01	7.45	0.057	14.90	14.62
	$CO(NH_2)_2$					
	0	0	0	0.018	1.03	1.01
	CH_3NH_2					
	0	0	0	0.0033	0.65	0.64
HCO_2^- NH_3	$HCONH_2$					
	0.0018	0.22	0.21	0.032	8.08	10.32
	CH_3CONH_2					
	0.0070	0.40	0.42	0.0056	5.97	7.64
CH_3COO^- NH_3	$HCONH_2$					
	0	0	0	0.032	3.56	3.10
	CH_3CONH_2					
	0.0028	0.24	0.29	0.021	9.65	8.42
CO_2 NO_3^{2-}	$HCONH_2$					
	0.0050	0.68	0.60	0.041	5.26	5.36
	CH_3CONH_2					
	0	0	0	0.0024	1.02	0.14
CO_2 N_2	$HCONH_2$					
	0	0	0	0.0078	1.56	0.98
	CH_3CONH_2					
	0	0	0	0.0021	0.92	0.58
CO_2 SO_3^{2-}	$CH_3O_3S^-$					
	0.0028	0.24	0.78	0.014	6.16	5.38

Note: We estimate nitrogen reactants and sulfur reactants based on the following principles:

$CO_2 + NO_3^{2-}$: 8 electrons for formamide production, 14 for acetamide (nitrate to NH_3 is 6 electrons)

$CO_2 + N_2$: 5 electrons for formamide, 11 for acetamide (each NH_3 is 3 electrons from N_2)

$CO_2 + SO_3^{2-}$: 6 electrons for methane sulfonate.

References

1 Chen, L., Ma, J., Huang, Y., Dai, M. H. & Li, X. L. Optimization of a colorimetric method to determine trace urea in seawater. *Limnol Oceanogr-Meth* **13**, 303-311 (2015). https://doi.org:10.1002/lom3.10026

2 Lv, C. D. *et al.* A Defect Engineered Electrocatalyst that Promotes High-Efficiency Urea Synthesis under Ambient Conditions. *Acs Nano* **16**, 8213-8222 (2022). https://doi.org:10.1021/acsnano.2c01956

3 Feng, Y. G. *et al.* Te-Doped Pd Nanocrystal for Electrochemical Urea Production by Efficiently Coupling Carbon Dioxide Reduction with Nitrite Reduction. *Nano Letters* **20**, 8282-8289 (2020). https://doi.org:10.1021/acs.nanolett.0c03400

4 O'Keeffe, M. & Sherington, J. Comparison of three methods for the determination of urea in compound feed and silage. *Analyst* **108**, 1374-1379 (1983). https://doi.org:10.1039/an9830801374

5 Zawada, R. J., Kwan, P., Olszewski, K. L., Llinas, M. & Huang, S. G. Quantitative determination of urea concentrations in cell culture medium. *Biochem Cell Biol* **87**, 541-544 (2009). https://doi.org:10.1139/o09-011

6 Wei, X. X. *et al.* Oxygen Vacancy-Mediated Selective C-N Coupling toward Electrocatalytic Urea Synthesis. *J Am Chem Soc* (2022). https://doi.org:10.1021/jacs.2c03452

7 Jiang, H. P., Zhang, Y. H., Yang, K. L. & Zou, J. Validation of a LC-MS Method for the Determination of Urea Contamination in Market Teas. *Food Anal Method* **7**, 13-20 (2014). https://doi.org:10.1007/s12161-013-9590-z

8 Wu, Y. S., Jiang, Z., Lin, Z. C., Liang, Y. Y. & Wang, H. L. Direct electrosynthesis of methylamine from carbon dioxide and nitrate. *Nat Sustain* **4**, 725-+ (2021). https://doi.org:10.1038/s41893-021-00705-7

9 Liler, M. Nuclear Magnetic Resonance Spectra of Acetamide N-15 - Solvent and Temperature Effects. *J Magn Reson* **5**, 333-& (1971). https://doi.org:Doi 10.1016/0022-2364(71)90084-9

10 Chen, C. *et al.* Coupling N2 and CO2 in H2O to synthesize urea under ambient conditions. *Nat. Chem.* **12**, 717-724 (2020). https://doi.org:10.1038/s41557-020-0481-9

11 Jouny, M. *et al.* Formation of carbon-nitrogen bonds in carbon monoxide electrolysis. *Nat Chem* **11**, 846-851 (2019). https://doi.org:10.1038/s41557-019-0312-z

12 Guo, C. Y. *et al.* Electrochemical Upgrading of Formic Acid to Formamide via Coupling Nitrite Co-Reduction. *J Am Chem Soc* (2022). https://doi.org:10.1021/jacs.2c05660

13 Hodgetts, R. Y. *et al.* Refining Universal Procedures for Ammonium Quantification via Rapid H-1 NMR Analysis for Dinitrogen Reduction Studies. *Acs Energy Letters* **5**, 736-741 (2020). https://doi.org:10.1021/acsenergylett.9b02812

14 Liu, L. Y., Mo, H. P., Wei, S. W. & Raftery, D. Quantitative analysis of urea in human urine and serum by H-1 nuclear magnetic resonance. *Analyst* **137**, 595-600 (2012). https://doi.org:10.1039/c2an15780b

15 Timoshenko, J. *et al.* Steering the structure and selectivity of CO2 electroreduction catalysts by potential pulses. *Nature Catalysis* **5**, 259-267 (2022). https://doi.org:10.1038/s41929-022-00760-z

16 Matsui, T. *et al.* In Situ Attenuated Total Reflection Infrared Spectroscopy on Electrochemical Ammonia Oxidation over Pt Electrode in Alkaline Aqueous Solutions. *Langmuir* **31**, 11717-11723 (2015). https://doi.org:10.1021/acs.langmuir.5b02330

17 Liu, Q. *et al.* High-Performance Electrochemical Nitrate Reduction to Ammonia under Ambient Conditions Using a FeOOH Nanorod Catalyst. *Acs Appl Mater Inter* **14**, 17312-17318 (2022). https://doi.org:10.1021/acsami.2c00436

18 Daiyan, R. *et al.* Nitrate reduction to ammonium: from CuO defect engineering to waste NOx-to-NH3 economic feasibility. *Energ Environ Sci* **14**, 3588-3598 (2021). https://doi.org:10.1039/d1ee00594d

19 Lu, X. *et al.* In Situ Observation of the pH Gradient near the Gas Diffusion Electrode of CO2 Reduction in Alkaline Electrolyte. *Journal of the American Chemical Society* **142**, 15438-15444 (2020). https://doi.org:10.1021/jacs.0c06779

20 Gupta, N., Gattrell, M. & MacDougall, B. Calculation for the cathode surface concentrations in the electrochemical reduction of CO2 in KHCO3 solutions. *J Appl Electrochem* **36**, 161-172 (2006). https://doi.org:10.1007/s10800-005-9058-y

21 Jeon, H. S. *et al.* Selectivity Control of Cu Nanocrystals in a Gas-Fed Flow Cell through CO2 Pulsed Electroreduction. *J Am Chem Soc* **143**, 7578-7587 (2021). https://doi.org:10.1021/jacs.1c03443
